# Mechanical observables of cancer invasion: actin, adhesion, and metastatic plasticity

**DOI:** 10.3389/fcell.2026.1930678

**Published:** 2026-09-03

**Authors:** Subhajit Dutta, Sushree Sulava

**Affiliations:** 1 Department of Biochemistry and Molecular Cell Biology (IBMZ), Center for Experimental Medicine, University Medical Center Hamburg-Eppendorf, Hamburg, Germany; 2 School of Biological Sciences, National Institute of Science Education and Research (NISER), Bhubaneswar, Odisha, India

**Keywords:** actin cytoskeleton, actin flow, cancer invasion, collective migration, extracellular matrix, focal adhesions, mechanotransduction, nematic order

## Abstract

Cancer invasion is commonly classified through epithelial-mesenchymal plasticity, yet cells with similar transcriptional states can differ substantially in force production, cytoskeletal organization and collective mobility. We critically examine whether mechanical measurements can supply information that is not already contained in molecular or histopathological classifications. The available evidence does not justify a universal five-dimensional phase space. It supports a more restrained hierarchy: primary measurements (filament orientation, cortical tension, substrate traction, cell and nuclear boundaries, and velocity fields), derived descriptors (polarity, field-specific normalized nematic order, shape and density metrics), and tissue-scale constructions (orientation fields, defects and inferred unjamming states). These levels are coupled and often mathematically dependent. We correct several common conflations. Traction-force microscopy recovers substrate traction through an inverse mechanical model; it does not directly measure intrinsic cortical active stress. Isotropic cortical contractility must be separated from anisotropic nematic stress. Structural actin order must likewise be distinguished from apolar alignment calculated from an optical-flow field; the latter is a dynamic image-derived descriptor, not a direct measurement of filament orientation or active stress. The vertex model threshold $p_{0}^{*}≃3.81$ is a model parameter, not a universal cutoff for segmented tumor cells. Patient-tissue unjamming maps instead use multivariate shape-density information, including cell-and-nucleus shape (CeNuS) and nuclear number density. We evaluate evidence supporting and opposing the independence of unjamming from epithelial-mesenchymal transition, the context-dependent effects of contractility, and the proposed causal role of topological defects. Our conclusion is deliberately conditional: mechanical observables are most credible as complementary, scale-specific measurements whose incremental value must be established against EMT/EMP state, tumor grade and tissue architecture in the same specimens. This formulation retains the explanatory power of mechanics while making its assumptions, dependencies and clinical limits explicit.

## 1 Cancer invasion as a mechanical-state problem

### 1.1 The clinical stakes and the diversity of invasion phenotypes

In solid cancers, local invasion and distant metastasis—rather than proliferation of the primary mass alone—account for most solid-tumor mortality (Gandalovičová et al., 2017). The events that kill patients are migratory and mechanical, executed by the actin cytoskeleton, yet the tools used to stratify tumors—grade, molecular subtype and gene-expression signatures—generally do not measure mechanics directly.

Tumors do not invade by a single mechanism. Invasion fronts show a diversity of actin-driven phenotypes: elongated mesenchymal cells with lamellipodia and stress-fiber traction; rounded amoeboid cells with cortical blebs and minimal adhesion (Wolf et al., 2003); collective strands that maintain junctions (Hegerfeldt et al., 2002) and distribute traction across many cells; tumor buds (a single cell or cluster of up to four cells detaching at the front, a clinically recognized colorectal feature; Lugli et al., 2017); cells co-migrating with cancer-associated fibroblasts (Gaggioli et al., 2007); and perineural invasion (Marcadis et al., 2023). A cell in dense pancreatic desmoplasia faces different physical constraints than one spreading along lung alveoli or a glioblastoma cell tracking white matter.

### 1.2 Plasticity: the transcriptional reading and its limits

Crucially, individual cancer cells are not locked into one phenotype: they switch, reversibly and rapidly. Friedl and Wolf cataloged this as a set of interconvertible migration modes and showed that blocking one mode (protease inhibition) triggers escape into another (the mesenchymal–amoeboid transition), so cells rescue motility by switching rather than stopping (Wolf et al., 2003; Friedl and Wolf, 2003). Their “multiscale tuning model” framed migration mode as the tunable output of molecular determinants—adhesion receptors, proteases, Rho-GTPase signaling—acting on the cytoskeleton (Friedl and Wolf, 2010).

The dominant contemporary framework casts this plasticity as primarily transcriptional. Epithelial–mesenchymal plasticity (EMP), the continuum between epithelial and mesenchymal states traversed via partial/hybrid intermediates, is driven by EMT-inducing transcription factors—SNAIL, ZEB1/2 and TWIST1 — that repress junctional genes and reprogram the cytoskeleton (Pastushenko and Blanpain, 2019). We should not caricature this literature: the mechanotransduction field has long understood the relationship runs both ways—YAP/TAZ allows mechanical state to feed back onto transcription (§3.5), and coupled-circuit models already treat EMT and the mesenchymal–amoeboid transition as a joint phase space (miR-200/ZEB coupled to Rac/RhoA circuit; Huang et al., 2015). What characterizes the dominant reading is therefore not a belief that the cytoskeleton is merely an effector, but a choice of *coordinates*: even bidirectional models place the axes of plasticity in gene-regulatory variables, with mechanics entering as consequence or feedback rather than as a primary coordinate.

That choice is powerful within its scope. The specific limitation is that at least one epithelial transition to collective motility is not simply a readout of a canonical EMT transcriptional axis. Measuring that transition mechanically can therefore capture behavior that a transcriptional classification alone may miss.

### 1.3 The dissociation: plasticity is also mechanical, not only transcriptional

The most direct evidence comes from the jamming-to-unjamming transition, a concept rooted in the broader physics of collective cell migration (Angelini et al., 2011; Ladoux and Mège, 2017; Alert and Trepat, 2020). Confluent epithelial collectives can occupy solid-like jammed or fluid-like unjammed regimes. The controlling variables depend on the model and system: the canonical vertex model links its target shape parameter to the competition between cortical tension and adhesion, whereas self-propelled models additionally depend on motility and persistence (Bi et al., 2015; Bi et al., 2016). These transitions therefore cannot be identified with epithelial-to-mesenchymal conversion alone. Crucially, when partial EMT and unjamming are triggered in the *same* primary human epithelial cells they remain distinct: after unjamming, junctions, apico-basal polarity and barrier function remain intact and mesenchymal markers stay unapparent, yet cells elongate, align and migrate cooperatively. A computational decomposition attributes partial EMT mainly to diminished junctional tension but unjamming mainly to augmented cellular propulsion—two mechanical routes to migration that can be separated from the canonical EMT axis (Mitchel et al., 2020).

The strength and limit of this evidence must be stated precisely. The cleanest demonstration that unjamming and EMT are mechanically and transcriptionally separable (Mitchel et al., 2020; De Marzio et al., 2021) was made in primary human bronchial epithelial cells, not cancer. The clearest cancer-model evidence that a collective can invade while retaining epithelial identity is from MCF10A.DCIS breast cells driven by RAB5A (Palamidessi et al., 2019), while compression-driven unjamming in breast epithelial/cancer models can occur without EMT (Cai et al., 2022). A complementary patient-tissue literature now shows that shape- and density-based unjamming or tissue-fluidity states exist in primary breast and cervix carcinomas, that jammed and unjammed regions can coexist within explants, and that predicted motility through unjamming correlates with distant metastasis in a large breast-cancer cohort (Grosser et al., 2021; Fuhs et al., 2022; Gottheil et al., 2023). These studies substantially strengthen the cancer relevance of unjamming, but they do not by themselves prove that the unjamming axis is transcriptionally independent in tumors, because EMT-transcription-factor state was not systematically measured in the same cells. Thus, the open question is narrower than before: unjamming-like mechanical states are present and prognostically relevant in cancer, but their independence from EMT/EMP programs in patient tissue requires direct validation.

The logical consequence is therefore not that transcriptional identity is irrelevant, but that it is incomplete. If a tumor-cell collective can become invasive while epithelial identity is retained, and if shape-/density-defined motility states can stratify tumor tissue, then mechanical variables may carry information that is not captured by molecular markers alone. How often that information is independent of, redundant with or coupled to transcription is the empirical question this Review is organized to pose.

### 1.4 From a putative coordinate system to a hierarchy of observables

The original attraction of a coordinate framework is clear: a small set of quantities could, in principle, compare invasion programs that use different molecular machinery. The evidence does not yet warrant treating those quantities as independent axes of a tumor phase space. Several are computed from the same images; some are constitutively related; and others are defined only after spatial coarse-graining. Calling all of them order parameters also obscures an important distinction between a formal variable in a model and a measured feature in a specimen.

We therefore organize the review as a hierarchy. At the first level are primary or near-primary measurements: filament and cell orientations, substrate displacements, cortical tension, cell and nuclear boundaries, and velocity or optical-flow fields. The second level comprises derived descriptors, including filament polarity P , structural actin order $S_{\text{actin}}$ , apolar flow alignment $S_{\text{flow}}$ , polar flow coherence $P_{\text{flow}}$ , shape index q , density, and traction moments. The third level contains tissue constructions—cell-orientation fields, topological defects and multivariate shape-density state diagrams. This hierarchy is not semantic housekeeping. It specifies what is observed, what is calculated, and where a constitutive model enters the inference.

Three judgments follow. First, the strongest cancer evidence currently concerns collective fluidization and shape-density states in breast and cervix tumors, not a complete actin-to-tissue coordinate map (Grosser et al., 2021; Fuhs et al., 2022; Gottheil et al., 2023). Second, the cleanest transcriptional dissociation between unjamming and EMT remains derived from airway epithelium, whereas cancer studies show context-dependent coupling between cell-cell adhesion, confinement and individualization (Mitchel et al., 2020; Ilina et al., 2020). Third, the tissue-scale active-nematic analogy is promising but remains least secure in heterogeneous three-dimensional tumors. The resulting synthesis is a measurement agenda, not a claim that cancer invasion has already been reduced to a universal state space.

One experimentally tractable bridge lies between filament architecture and whole-cell migration: live image-derived actin motion. Dense velocity estimation can quantify alignment, polarity, deformation and temporal persistence within individual cells. These descriptors are attractive because they retain dynamics that fixed cytoskeletal images lose. Their interpretation must nevertheless remain exact: order in an estimated flow field is neither direct filament order nor force, and separation of cell lines is not equivalent to prediction of metastatic behavior. This distinction defines both the opportunity and the validation burden.

### 1.5 Why the distinction matters for therapy and prognosis

Migration plasticity is therapeutically relevant because inhibition of one route may redirect cells into another. Protease blockade can favor protease-independent amoeboid migration, and inhibition of one actin nucleator can be buffered by another (Wolf et al., 2003; Dimchev et al., 2021). These observations support the migrastatic principle, but they do not show that a mechanical classifier will predict treatment response. The clinically defensible question is narrower: does a mechanically derived feature improve prediction after established variables—stage, grade, subtype, stromal content and EMT/EMP state—have been included?

The distinction also guards against target-level overinterpretation. Contractility can promote squeezing, matrix remodeling and survival under stress, but non-muscle myosin IIA can act as a tumor suppressor through parallel effects on p53 stability (Schramek et al., 2014). E-cadherin loss can facilitate individualization in some settings, whereas epithelial cohesion and E-cadherin can be required for metastatic dissemination in others (Ilina et al., 2020; Padmanaban et al., 2019). Mechanical analysis must accommodate such reversals rather than assign every regulator a fixed pro- or anti-invasive direction. Box 3 examines the corresponding problem for single-target migrastatic strategies.

## 2 Coupled mechanical observables across scale

**FIGURE 1:**
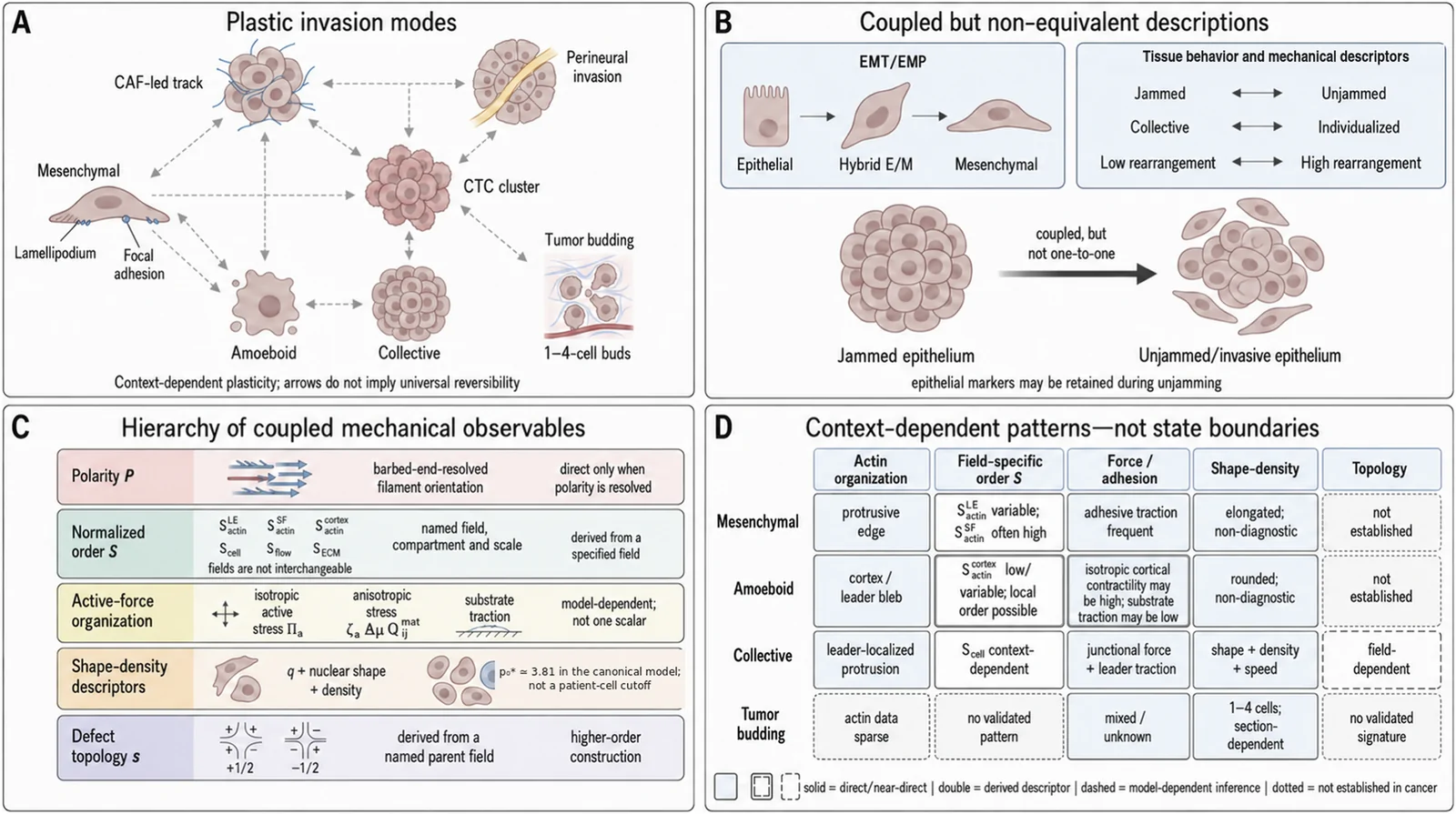
Coupled mechanical observables and context-dependent invasion patterns. **(A)** Cancer invasion phenotypes are connected by context-dependent plasticity; arrows do not imply universal reversibility. **(B)** EMT/EMP status and tissue behavior are coupled but non-equivalent, and epithelial markers can be retained during unjamming. **(C)** The proposed framework is a hierarchy rather than a set of independent coordinates: polarity requires a resolved polar field; normalized orientational order must specify the source field, compartment and scale; active-force organization separates isotropic cortical contractility, anisotropic stress and substrate traction; shape-density descriptors remain context dependent; and defects are derived from a named parent field. The canonical vertex-model target-shape threshold is model specific and is not a patient-cell cutoff. **(D)** Mesenchymal, amoeboid, collective and budding phenotypes therefore show conditional patterns, not validated state boundaries. Line styles distinguish direct or near-direct measurement, derived descriptors, model-dependent inference and relationships not established in cancer.

The quantities discussed below are not independent coordinates. They are measurements and derived descriptors at different spatial scales, connected by constitutive assumptions and shared image features (Figure 1). We retain the symbols because they are useful, but we specify the field, compartment, dimensionality and evidence level whenever a value is reported. Box 1 gives the formal definitions.

### 2.1 Filament polarity (*P*): a local descriptor with difficult tissue access

For an actin filament with barbed-end direction t and a chosen reference direction m , a local polar order can be written $P=\langle t\cdot m\rangle =\langle \cos\theta \rangle$ . Its range is −1 to 1, and the sign changes when filament orientation reverses. The definition requires barbed-end identity; an unoriented fluorescence texture cannot provide it. Cryo-electron tomography can resolve filament polarity in restricted specimens, whereas quantitative fluorescent speckle microscopy and optical flow report actin transport or coherence, not barbed-end polarity itself. Those dynamic measurements are informative proxies but also contain polymerization-, depolymerization- and myosin-driven motion.

Arp2/3-mediated branching, formins, Ena/VASP, capping proteins and cofilin control the spatial organization of protrusive networks (Pollard and Borisy, 2003; Svitkina, 2018). Their redundancy is biologically important: Arp2/3 depletion can induce FMNL2/3-dependent filopodial migration rather than abolish protrusion (Dimchev et al., 2021). The inference that a tumor retains P through a nucleator switch is mechanistically plausible, but a formal P has rarely been measured in cancer tissue. We therefore use P only for local, polarity-resolved actin data and do not infer it from cell shape alone.

The molecular basis for this interpretation is broader than Arp2/3 alone. Polymerization-ratchet models established how biased filament assembly can generate protrusive force (Mogilner and Oster, 1996), and structural work showed how Arp2/3-generated branches orient daughter filaments toward the membrane (Rouiller et al., 2008). Acute Arp3 perturbation indicates that Arp2/3 organizes the spatial distribution of capping protein and cofilin rather than simply setting a bulk polymerization rate (Koestler et al., 2013). Formin-family effects are context-dependent: DIAPH1 loss perturbs innate-immune signaling in myelodysplasia (Keerthivasan et al., 2014), whereas DIAPH3 loss destabilizes microtubules and promotes amoeboid blebbing in defined carcinoma models (Hager et al., 2012). Cofilin-pathway activity, rather than expression of one component, controls mammary-tumor invasion (Wang et al., 2006), while fascin-1 overexpression is associated with adverse outcome across several carcinomas (Tan et al., 2013). The shared monomer pool adds a further layer: cyclase-associated protein and cofilin recharge actin monomers (Kotila et al., 2019), and reduced profilin-1 can increase breast-cancer invasiveness (Zou et al., 2007). These findings support redundant control of protrusion but do not establish a formal P value in patient tissue.

### 2.2 Normalized nematic order (*S*): field and compartment must be named

For a two-dimensional head-tail-symmetric orientation field,

$$Q_{ij}^{\left(2D\right)}=\langle n_{i}n_{j}-\frac{1}{2}\delta_{ij}\rangle .$$

The largest eigenvalue of this tensor ranges from 0 to 1/2 . A scalar with the conventional range 0–1 is therefore

$$S_{2D}=2\lambda_{\max}\left(Q^{\left(2D\right)}\right)=\left|\langle e^{2i\theta }\rangle \right|.$$

For a three-dimensional uniaxial field,

$$Q_{ij}^{\left(3D\right)}=\langle n_{i}n_{j}-\frac{1}{3}\delta_{ij}\rangle ,\;S_{3D}=\frac{3}{2}\lambda_{\max}\left(Q^{\left(3D\right)}\right).$$

This scalar also ranges from 0 to 1 for isotropic-to-perfect uniaxial order. Strongly biaxial fields require the full eigenvalue spectrum; one scalar is insufficient.

The mathematical form does not identify the physical field. When a dense optical-flow algorithm estimates a velocity vector $v\left(x,t\right)$ , an apolar flow-alignment tensor can be defined as

$$Q_{ij}^{\text{flow}}=\frac{\langle w\left({\hat{v}}_{i}{\hat{v}}_{j}-\delta_{ij}/2\right)\rangle }{\langle w\rangle },\;S_{\text{flow}}=2\lambda_{\max}\left(Q^{\text{flow}}\right),$$

where w is a declared reliability or intensity weight. $S_{\text{flow}}$ measures alignment of estimated motion after the sign of velocity has been discarded. It is not direct filament orientation, barbed-end polarity, cortical tension or active stress. Because a velocity field is intrinsically polar, an apolar projection should be accompanied by a polar coherence statistic,

$$P_{\text{flow}}=\left|\frac{\langle w\hat{v}\rangle }{\langle w\rangle }\right|,$$

which distinguishes unidirectional coherent motion from equal, oppositely directed streams that can have high $S_{\text{flow}}$ but low $P_{\text{flow}}$ . Optical-flow estimates additionally depend on brightness-constancy, regularization, mask and temporal-sampling assumptions (Horn and Schunck, 1981; Zach et al., 2007). Agreement across algorithms can establish robustness of the image-derived statistic, but cannot by itself validate its biological interpretation.

The field must be stated explicitly. We distinguish $S_{\text{actin}}^{LE}$ at the branched leading edge, $S_{\text{actin}}^{SF}$ in stress fibers, $S_{\text{actin}}^{cortex}$ at the cortex, $S_{\text{cell}}$ for cell-body axes, $S_{\text{flow}}$ for apolar alignment of actin-associated motion, $P_{\text{flow}}$ for polar flow coherence, $S_{\text{vel}}$ for cell-velocity directions and $S_{\text{ECM}}$ for stromal fibers. These variables are not exchangeable. A mesenchymal cell can simultaneously have high protrusive polarity, low local leading-edge nematic order and highly aligned stress fibers. A cobblestone epithelium may have low or poorly defined $S_{\text{cell}}$ ; elongation and collective alignment during unjamming can increase it. Thus, neither “mesenchymal = high $S_{\text{actin}}$ ” nor “epithelial = high $S_{\text{cell}}$ ” is generally valid without compartment and scale. When cell eccentricity approaches zero, the major-axis angle is statistically ill-defined even if a segmentation algorithm returns a value. Cell-body order should therefore be reported with an axis-confidence or eccentricity criterion, and conclusions should be tested with low-confidence cells excluded or down-weighted. Dynamic order also requires a temporal descriptor: an instantaneous or time-averaged $S_{\text{flow}}$ does not report persistence, switching or relaxation. Autocorrelation time, state dwell time and early-versus-late estimates should be reported separately and calculated with the cell, not the image frame, as the biological unit of replication.

Mechanistic precedent for structural order should also be retained without overextending it. Concentrated reconstituted actin can undergo Onsager-type nematic alignment (Käs et al., 1996), but this does not prove that a cancer-cell cortex is in the same thermodynamic regime. Bleb nucleation depends on local membrane-cortex detachment and pressure (Charras and Paluch, 2008); ERM proteins contribute to cortical attachment and organization (Fehon et al., 2010); and Eps8 and Arp2/3 can organize leader-bleb actin while supporting cortical tension (Logue et al., 2015). Thus a rounded cell may contain locally ordered actin structures even when its global cortical nematic order is low.

### 2.3 Active-force organization: isotropic and anisotropic components

A traceless nematic term cannot represent isotropic cortical contraction. The minimal decomposition is

$$\sigma_{ij}^{\text{active}}=-\Pi_{a}\delta_{ij}+\zeta_{a}\Delta \mu \;Q_{ij},$$

where $\Pi_{a}$ denotes isotropic active cortical pressure or tension and $\zeta_{a}\Delta \mu Q_{ij}$ the anisotropic active stress associated with a specified orientation field. Sign conventions for $\zeta_{a}$ differ across the active-matter literature and must be declared for each model. Under the convention written here, $\Pi_{a}>0$ contributes an isotropic contractile stress. It is an effective coarse-grained stress, not automatically identical to a membrane-tension measurement or to a Laplace pressure unless the appropriate geometry and constitutive assumptions are supplied. More importantly, $\Pi_{a}$ , $\zeta_{a}$ , $Q_{ij}$ and substrate traction are not different names for one scalar “contractile state.”

This distinction resolves the apparent paradox of rounded amoeboid cells. They may have high isotropic cortical contractility ( $\Pi_{a}$ ) while their global actin nematic order is low, so the anisotropic term is small. A mesenchymal cell may show strong traction and aligned stress fibers while retaining a branched, locally disordered leading edge. RhoA-ROCK-myosin, Rac1-WAVE-Arp2/3, adhesion and confinement regulate these components, but no single sign reliably identifies a migration phenotype across substrates and geometries.

Traction-force microscopy reconstructs forces exerted on a deformable substrate from measured displacements, a substrate constitutive model, boundary conditions and regularized inversion. It therefore measures substrate traction in a model-dependent manner; it does not directly recover the sign or magnitude of intrinsic cortical active stress (Zancla et al., 2022). Cortical-force organization should instead be triangulated from traction, cortical tension or aspiration, RhoA/Rac1 activity, pMLC, adhesion architecture and live morphology. We use “active-force organization” for this multicomponent description and avoid the former cell-averaged scalar σ . The same restriction applies to image-derived activity indices. A product such as $S_{\text{flow}}\left|v\right|$ can be useful as a kinematic summary, but it is not an active-stress measurement and cannot replace $\Pi_{a}$ or $\zeta_{a}\Delta \mu Q_{ij}$ . Correlation with strain rate is evidence of kinematic coupling; constitutive stress identification requires independent force or tension measurements and an explicit mechanical model.

### 2.4 Shape, density and jamming: three quantities that must not be collapsed

For a segmented two-dimensional cell, $q=P_{\text{cell}}/\sqrt{A_{\text{cell}}}$ is a dimensionless geometric feature. In the canonical confluent two-dimensional vertex model, the rigidity transition occurs near the *target* shape parameter $p_{0}^{*}≃3.81$ under the assumptions of that model (Bi et al., 2015). $p_{0}$ is a parameter in the energy functional; it is not identical to the observed shape index of an individual tumor cell. In self-propelled Voronoi models, the transition also depends on motility and persistence (Bi et al., 2016). Density, adhesion, heterogeneity, proliferation, three-dimensional geometry and matrix coupling further alter the relationship.

Experimental work nevertheless establishes that cell and nuclear shape can report collective fluidity in defined systems (Park et al., 2015; Grosser et al., 2021). Breast-cancer invasion in three-dimensional matrices is governed jointly by cell-cell adhesion and available extracellular space rather than by a single shape cutoff (Ilina et al., 2020). Most importantly, the patient state diagram of Gottheil et al. (2023) did not classify tumors with q=3.81 . It combined cell-and-nucleus shape (CeNuS) with standardized mean cell area as a proxy for inverse nuclear number density and calibrated the static morphology against motility in living explants. This multivariate, cohort-derived construction is the relevant clinical precedent.

Accordingly, we separate: (i) $p_{0}^{*}$ as a model-specific transition parameter; (ii) measured q , nuclear aspect ratio and density as structural features; (iii) a calibrated multivariate unjamming classifier; and (iv) a clinically validated prognostic test. Evidence is substantial for (i)-(iii) in selected systems, but external prospective validation of (iv) and head-to-head comparison with molecular predictors remain necessary.

### 2.5 Tissue orientation fields and topological defects

Topological defects are derived from an orientation field; they are not independent measurements. For a two-dimensional nematic director, $s={\left(2\pi \right)}^{-1}\oint d\theta$ , with common charges +1/2 and −1/2 . If the doubled phase ϕ=2θ is used computationally, the equivalent expression is $s={\left(4\pi \right)}^{-1}\oint d\phi$ ; a half-charge defect therefore corresponds to a ±2π winding of ϕ , not ±π . Defect position and charge depend on the field definition, smoothing scale, segmentation and boundary handling. The parent field must be named: $s_{\text{actin}}$ , $s_{\text{flow}}$ and $s_{\text{cell}}$ describe singularities in structural actin, an apolarized motion field and a cell-body orientation field, respectively, and need not coincide. In particular, a defect in $Q_{\text{flow}}$ is a defect of a derived line field obtained after discarding velocity sign; it does not by itself demonstrate that the material is an active nematic. In MDCK monolayers, +1/2 defects have been associated with compressive stress, YAP activation and extrusion, whereas −1/2 defects show different stress organization (Saw et al., 2017). This is causal evidence in a tractable epithelial model, not yet a general rule for tumors.

Even the relevant symmetry can depend on scale. Confluent epithelia can show a crossover from local hexatic organization to nematic organization at larger length scales (Armengol-Collado et al., 2023; Eckert et al., 2023). Consequently, a defect catalogue is meaningful only after the symmetry class, coarse-graining scale and parent field have been justified.

Cancer evidence is more limited. Confined collective migration in mouse dermis displays active-matter-like flow statistics (Chepizhko et al., 2025), but such statistics do not establish defect-mediated invasion. Emerging three-dimensional tumor studies are intriguing but remain insufficient to assign a universal biological function to defect charge. At present, defect maps should be treated as higher-order descriptors whose predictive and causal value must be tested independently of geometry, density, proliferation and cell death.

### 2.6 Dependence, identifiability and the appropriate claim

The hierarchy contains explicit dependencies. $S_{\text{cell}}$ and q can be derived from the same segmentation; a defect map is calculated from its declared parent field; $S_{\text{flow}}$ , strain rate, divergence and vorticity can all be calculated from the same velocity estimate; anisotropic active stress contains a material $Q_{ij}$ by construction; and polarity and structural actin order can share the same filament data. A $Q_{\text{flow}}$ constructed from motion is not automatically the material $Q_{ij}$ in the active-stress constitutive equation. Correlation among these quantities is therefore expected even without a distinct biological coupling. A proposed cancer classifier must quantify covariance, measurement error and incremental information rather than count related transformations of the same data as independent evidence.

The defensible claim is consequently modest but useful: coupled mechanical observables can describe aspects of invasion that molecular labels do not measure directly. Whether they add independent biological or clinical information is an empirical question to be answered by multimodal, same-specimen studies.

### 2.7 Evidence taxonomy and limits on numerical claims

Numerical precision must not be mistaken for biological validation. We use five evidence classes throughout this review: formal definition, model-specific parameter or threshold, direct or near-direct measurement, indirect surrogate or model-based inference, and clinically evaluated variable. These classes are not interchangeable. A formal range defines mathematical admissibility; it does not define a cancer state. A model threshold is transferable only after its assumptions and calibration have been demonstrated in the target system. A surrogate requires validation against the quantity it is claimed to represent. A clinical association does not become a clinically validated test without reproducible measurement, external validation and prospective evidence of added value.

In this taxonomy, P , S , q and s are formal descriptors; $p_{0}^{*}$ is a model-specific threshold; membrane-resolved boundaries, resolved filament orientations, substrate displacements and tracked trajectories are direct or near-direct measurements; optical-flow fields are algorithm-dependent estimates from image intensity; $S_{\text{flow}}$ and $P_{\text{flow}}$ are derived from those estimates; and pMLC, nuclear shape, H&E-inferred cell boundaries and morphology-based force labels are surrogates for the mechanical quantities they are often claimed to represent. CeNuS-density is a clinically evaluated, cohort-associated construction that still requires external prospective validation. The text, tables and figures preserve these distinctions rather than treating all reported values as experimentally validated state boundaries.

## 3 Molecular control of mechanical observables

**FIGURE 2:**
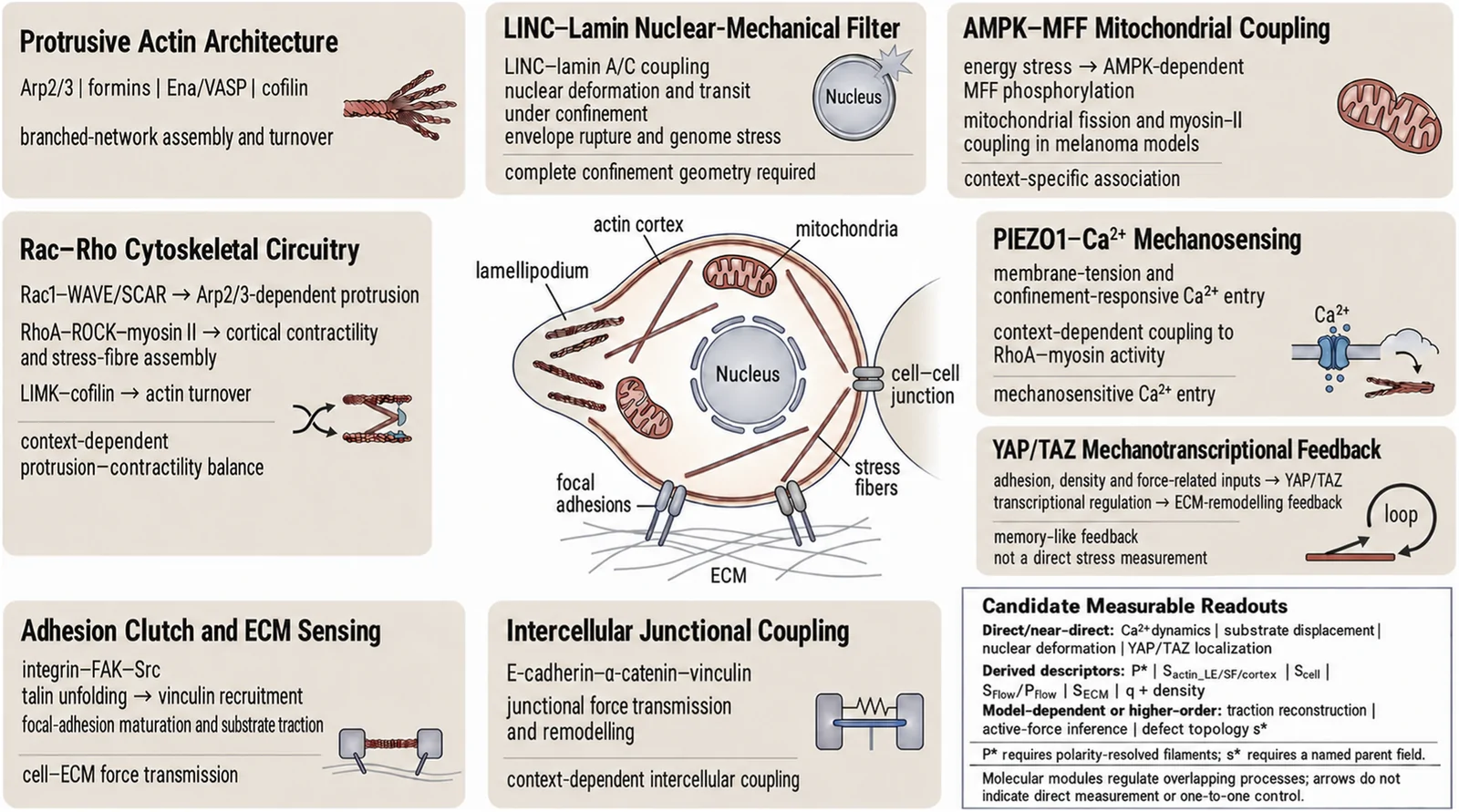
Molecular modules coupled to mechanically relevant processes and measurable readouts. The schematic summarizes pathways that can regulate protrusive actin architecture, Rac-Rho contractility balance, adhesion-clutch function, junctional coupling, nuclear mechanics, mitochondrial coupling, PIEZO1-Ca2+ mechanosensing and YAP/TAZ feedback. These modules are pleiotropic regulators, not one-to-one measurements of mechanical state. Candidate readouts are separated by measurement status: Ca2+ dynamics, substrate displacement, nuclear deformation and YAP/TAZ localization are direct or near-direct observations; polarity and field-specific orientational order are derived descriptors; and traction reconstruction, active-force inference and defect topology require inverse models or higher-order construction. Polarity requires a resolved polar field, defects require a named parent field, and arrows indicate regulation rather than direct measurement.

Mechanical observables are generated by molecular systems, not alternatives to them. The useful question is therefore not which gene “sets a coordinate” in isolation, but which measurements change after perturbation and whether that relation survives a change of substrate, dimensionality or cell state. The modules below are well established; their reduction to a universal mechanical state is not. Figure 2 summarizes these regulatory relationships.

### 3.1 Rho-GTPase circuits organize protrusion and contractility

Rac1 and RhoA coordinate protrusive and contractile programs through mutual antagonism, spatial feedback and downstream WAVE/Arp2/3, ROCK-myosin and adhesion pathways (Ridley and Hall, 1992; Hall, 1998; Ridley, 2001; Etienne-Manneville and Hall, 2002; Lawson and Ridley, 2018; Crosas-Molist et al., 2022). Melanoma provides the strongest evidence that this circuit supports switching between elongated and rounded invasion modes (Sanz-Moreno et al., 2008). Mathematical and experimental work also supports bistability and hysteresis in defined models (Byrne et al., 2016). These results justify a state-switch interpretation within those systems, but not a universal contractile-versus-extensile scalar for all carcinomas.

RhoA activity can raise isotropic cortical tension, organize anisotropic stress fibers, or both; the outcome depends on adhesion and geometry. Rac1 can generate a polarized leading edge without lowering contractility elsewhere in the cell. The JAK1/STAT3-ROCK loop further couples actomyosin activity to redox protection and an immunomodulatory secretome (Sanz-Moreno et al., 2011). Conversely, MYH9 loss promotes squamous-cell carcinoma partly through p53 destabilization (Schramek et al., 2014). Contractility is therefore neither uniformly pro-invasive nor adequately captured by one readout. At minimum, studies should report Rho-GTPase activity, cortical pMLC distribution, adhesion state and compartment-specific actin organization. Clinical translation is additionally constrained by microenvironmental effects: ROCK inhibition can alter antitumor immunity as well as tumor-cell contractility (Barcelo et al., 2023).

### 3.2 The LIMK–cofilin convergence

Cofilin-1 activity is a convergence point of both Rho-GTPase branches and thus a shared regulatory convergence point for *P* and invadopodial turnover: ROCK→LIMK2 and PAK→LIMK1 both phosphorylate cofilin-1 at Ser3 (inactivating it), while SSH1/chronophin reactivate it (Crosas-Molist et al., 2022). Cofilin phosphorylation regulates actin turnover and invadopodial maturation, but it does not by itself define a formal polarity gradient. LIMK1 is enriched in malignant breast tissue, and experimental LIMK1 expression enhances breast-cancer-cell invasion through cofilin-associated signaling (McConnell et al., 2011). Its broader cellular functions make target-level interpretation more complex than inhibition of one invasion route. At invadopodia, cortactin (CTTN/EMS1), a candidate target within recurrent 11q13 amplicons in breast and head-and-neck carcinomas (Schuuring, 1995) sequesters cofilin-1; Src-mediated cortactin phosphorylation releases it, triggering four-stage maturation (precursor → cofilin-driven polymerization → stabilization → MT1-MMP-dependent degradation; Oser et al., 2009), with NHE1-dependent pH elevation providing a parallel release route (Magalhaes et al., 2011).

That proteolytic model has been productively complicated: melanoma cells invade 3D collagen without proteolysis by “bleb-driven worrying” — rapid (∼20 s) bleb cycles whose large blebs mechanically abrade collagen, with front adhesion–collagen engagement driving bleb enlargement through branched-actin polymerization (Driscoll et al., 2024). This is a third invasion mode that separates the mechanical from the proteolytic function of protrusive structures—evidence that a blebbing morphology does not specify a unique molecular or mechanical route.

### 3.3 Stress fibers, focal adhesions and the integrin-FAK-Src clutch

Adhesion is not an auxiliary annotation to active force; it determines how intracellular force is transmitted. Stress fibers differ in architecture and mechanical function, while adhesion maturation follows a force-dependent sequence involving integrin clustering, FAK/paxillin, talin unfolding, vinculin recruitment and reinforcement by zyxin, alpha-actinin and tensin (del Rio et al., 2009; Elosegui-Artola et al., 2017). The clutch relation between retrograde actin flow and substrate traction is biphasic rather than monotonic (Gardel et al., 2008; Craig et al., 2015). A cell can therefore show rapid actin flow with weak traction, high traction with slower flow, or strong cortical contractility with little substrate coupling.

This non-equivalence is mechanistically important. TFM-derived traction cannot be used as a direct measure of $\Pi_{a}$ or $\zeta_{a}\Delta \mu Q_{ij}$ ; nor does high pMLC prove efficient force transmission to matrix. A mechanistic account requires at least three layers: active-force generation, cytoskeletal organization and clutch engagement. Integrins also act context-dependently: α5β1 recycling can sustain forward movement, whereas altered integrin signaling can favor anchorage-independent survival. In squamous-cell carcinoma, tumor-initiating cells depend on α6β1 and FAK-Src signaling for invasion (Schober and Fuchs, 2011).

Adhesion further links invasion to cell-cycle control. Integrin signaling contributes to centrosome separation and cytokinetic abscission; altered adhesion can shift division toward tension-, septin- and Ras-dependent control and thereby affect genomic integrity (Kamranvar et al., 2016; Gupta et al., 2018; Gupta et al., 2019; Kamranvar et al., 2022a; Kamranvar et al., 2022b; Rani et al., 2022). These effects belong to a downstream consequence layer, not to the mechanical-observable set itself.

### 3.4 Cadherin mechanosensing and the intercellular clutch: junctional control of shape and orientation

The force-transmission problem at cell–cell junctions mirrors the integrin clutch and can influence tissue-scale shape, orientation and rearrangement. The molecular biology is well established: the E-cadherin–α-catenin complex binds actin under tension; α-catenin’s force-dependent conformational change exposes a vinculin-binding site, recruiting vinculin as a mechanical reinforcer (parallel to talin; le Duc et al., 2010), building a network that coordinates contractile oscillations and suppresses junctional Rac1-driven lamellipodia. The consequences for tissue morphology are less secure. Downregulation of E-cadherin, weakening of α-catenin or disruption of junctional vinculin impairs intercellular force transmission, but it does not determine the direction of change in cell-body order. An isotropic cobblestone region may have low $S_{\text{cell}}$ , whereas elongation and alignment during unjamming can increase it. Junctional perturbation may also change the rearrangement rate and defect landscape. Direct topological evidence is currently limited to non-cancer monolayers, in which α-catenin knockdown in MDCK cells increases defect density and extrusion (Saw et al., 2017). Whether this relation holds in carcinoma—where CDH1 methylation or SNAIL/ZEB1 repression can dismantle the clutch—remains untested. A direct test would perturb one EMT-relevant junctional event and measure $S_{\text{cell}}$ , q , rearrangement and defect density in the same tissue (§7).

### 3.5 YAP/TAZ: the memory-like mechanism that couples mechanics back to identity

YAP/TAZ makes the mechanical and transcriptional readings inseparable. The forward path is canonical—ECM stiffness → integrin clustering → FAK/Src → actomyosin tension → YAP nuclear translocation → pro-invasive transcription (CTGF, CYR61, AREG). The key point is the *loop*: active YAP promotes collagen-I alignment and tissue tension that further activate YAP; active YAP is detectable in invading breast cancer cells in patients and is required for collective invasion in 3D collagen and *in vivo* (Khalil et al., 2024), and LOX-mediated cross-linking (Levental et al., 2009) feeds the same loop. YAP/TAZ can therefore act as a transcriptional memory-like link between active-force organization and $S_{\text{ECM}}$ : once force activates it, it rewires gene expression to sustain the environment that activated it—a mechanistic reason why mechanics and transcription cannot be treated as independent causal layers.

### 3.6 Mechanosensing beyond adhesions: PIEZO1

The mechanosensitive channel PIEZO1, gated by membrane deformation from osmotic pressure, shear, stiffness, and confinement, transduces force into $Ca^{2+}$ influx and thence Rho-GTPase modulation, myosin regulation, and cytoskeletal remodeling (Dombroski et al., 2021). In breast cancer, changes in cell shape drive PIEZO1-dependent $Ca^{2+}$ influx that links cell geometry to EMP-associated signaling (So et al., 2024) — a non-adhesion route by which geometry can alter contractility and protrusive organization. Whether PIEZO1 in tumor-infiltrating lymphocytes allows matrix mechanics to modulate cytotoxic function remains unresolved, but it provides a testable link between mechanosensing and the immune microenvironment (§4.3).

### 3.7 Nuclear mechanics: a geometry-dependent filter

The nucleus is frequently the rate-limiting structure during migration through narrow three-dimensional spaces. The relevant geometry must be reported explicitly: channel width, height, cross-sectional area, constriction length, nuclear dimensions and matrix deformability. Davidson et al. (2014) tested constrictions 15, 5, 3 or 2 μm wide and 5 μm high; Bell et al. (2022) used 5-μm-high channels with widths from 1 to 15 μm. These experiments do not establish a universal 7-μm threshold.

Lower lamin A/C can increase nuclear deformability and accelerate transit through selected constrictions, whereas excessive nuclear softness can compromise survival. Constricted passage can rupture the nuclear envelope and generate DNA damage, with ESCRT-III-dependent repair limiting injury (Denais et al., 2016; Raab et al., 2016). The route through confinement is not uniquely contractile. Cells may increase actomyosin force, reduce lamin A/C, use adhesion-based traction, degrade matrix, exploit pre-existing tracks, or widen the path. Which mechanism dominates depends on the ratio between nuclear size and the complete pore geometry, the mechanical properties of the matrix and the time available for adaptation.

Migration-induced DNA damage should not be conflated with a demonstrated route to a specific rearrangement class. Mitotic microhomology-mediated break-induced replication can generate chromoanasynthesis (Ngo et al., 2026), but that mechanism has not yet been causally connected to constriction-induced nuclear-envelope rupture in tumors.

It is therefore appropriate to say that severe confinement can bias some cells toward contractility-dependent migration, not that a given pore size selects a universal active-stress sign. Direct evidence requires simultaneous measurement or perturbation of RhoA/ROCK-myosin activity, force transmission and nuclear transit in the stated geometry.

### 3.8 WAVE/SCAR feedback and invadopodial scaffolding

WAVE/SCAR activation—a principal regulator of Arp2/3-dependent lamellipodial architecture—is modulated at three cascade positions: upstream Rac1 competition by CYRI-B (Yelland et al., 2021); NHSL1-Scar/WAVE binding that dampens Arp2/3 activity (Law et al., 2021); and post-activation Abi phosphorylation tuned by adhesion and osmotic stress (Singh et al., 2021), together enabling graded branching control. Invadopodial scaffolding adds the proteolytic arm: Tks5 scaffolds invadopodia via Src-dependent $PI\left(3,4\right)P_{2}$ binding (Courtneidge, 2012); MT1-MMP delivery requires aPKCι–cortactin–dynamin-2 trafficking and Protrudin-mediated ER–endosome contacts (Rossé et al., 2014; Pedersen et al., 2020); and invadopodia act as docking sites coupling exosome secretion to matrix degradation (Hoshino et al., 2013).

### 3.9 The mechano-metabolic axis: AMPK and contractile persistence

A reinforcing coupling links cortical mechanics to mitochondrial dynamics in melanoma models. Reduced adhesion and traction are associated with lower ATP availability and increased AMP:ATP or ADP:ATP ratios, which activate AMPK. AMPK-dependent MFF phosphorylation promotes mitochondrial fission, while MYPT1 inactivation sustains myosin-dependent contractility. AMPK inhibition reduced metastatic behavior *in vivo*, and a corresponding mitochondrial/AMPK state was detected in disseminating regions of human tumors (Crosas-Molist et al., 2023). AMPK-driven phosphorylation of the ER-anchored formin INF2 promotes mitochondrial fission (Ding et al., 2024), and INF2 itself participates in cancer-cell migration and shape control (Heuser et al., 2018); related observations in ovarian (AMPK-dependent mitochondrial trafficking to the leading edge; Cunniff et al., 2016) and prostate cancer (syntaphilin loss freeing mitochondria for cortical redistribution; Caino et al., 2017) suggest mechano-metabolic feedback may be a general stabilizer of contractile migration, but systematic validation outside melanoma is lacking; Section 7 specifies the corresponding test.

## 4 The mechanical microenvironment: constraints, cues and reciprocal remodeling

**FIGURE 3:**
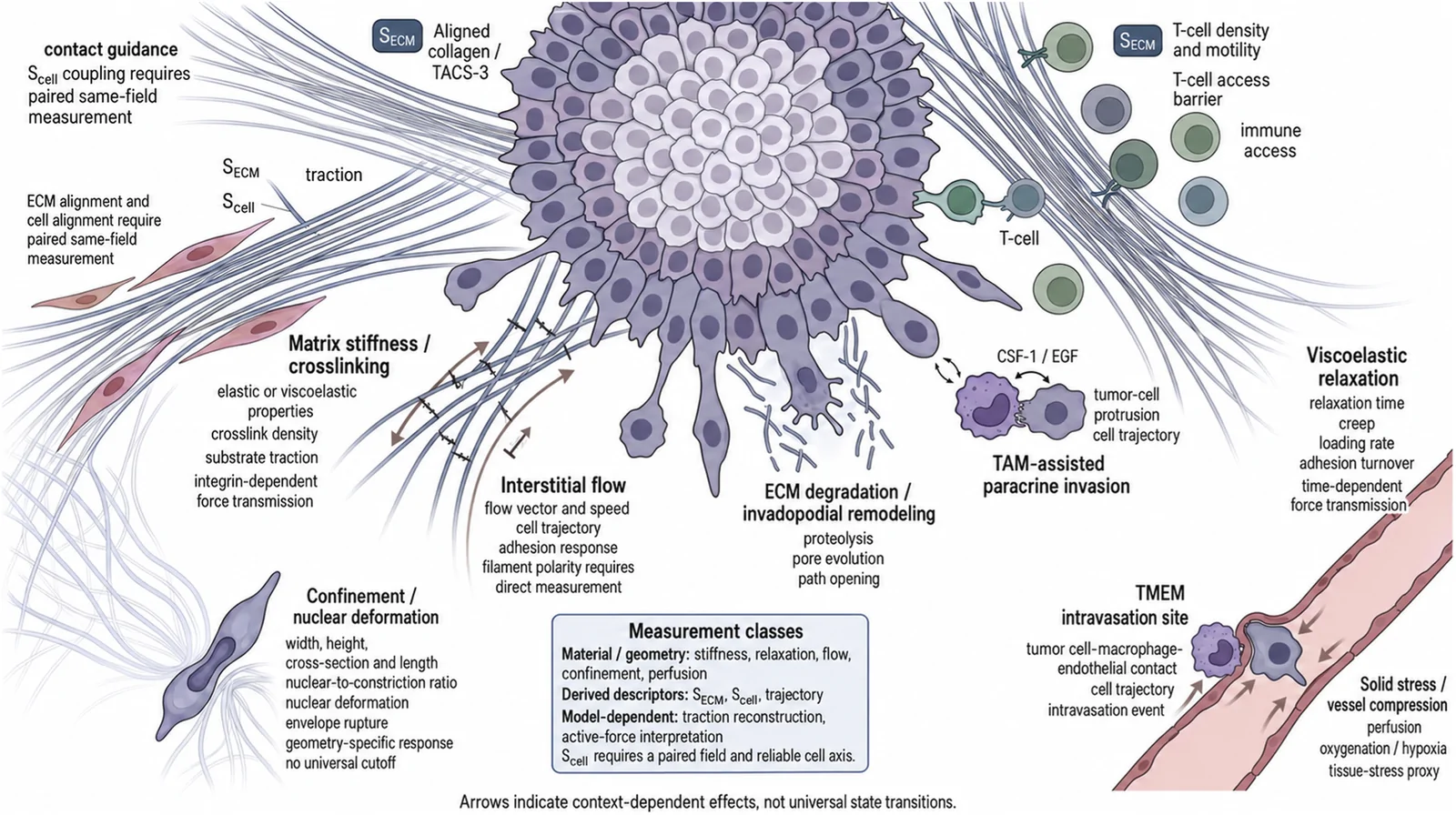
Microenvironmental constraints and candidate measurements in cancer invasion. Aligned collagen, CAF-generated tracks, matrix stiffness and crosslinking, interstitial flow, confinement, viscoelastic relaxation, ECM degradation, macrophage-assisted invasion, immune access and solid stress alter the mechanical options available to invading cells. ECM and cell-body alignment descriptors require named fields and paired same-field acquisition; traction is reconstructed from substrate deformation; and confinement must be reported through complete geometry, including width, height, cross-section, length and the nuclear-to-constriction ratio. The arrows indicate context-dependent effects, not universal state transitions, and active-force interpretation remains model dependent.

The microenvironment constrains which migration programs are mechanically accessible, while tumor cells reciprocally remodel those constraints. Figure 3 summarizes these reciprocal constraints, and Table 1 distinguishes their direct measurements from mechanistic inferences. We first examine physical cues, then the contributions of fibroblasts, macrophages and the immune interface.

**TABLE 1:** Mechanical cues, measurements and evidence level.

Cue	Reported biological response	What is measured	Interpretation and evidence level
Collagen alignment	Contact guidance; oriented collective or single-cell invasion	$S_{\text{ECM}}$ , fiber angle, TACS pattern	Direct stromal measurement; coupling to $S_{\text{cell}}$ must be tested in the same field
Matrix stiffness	Focal-adhesion maturation, YAP/TAZ activation, altered invasion	Elastic/viscoelastic material properties, traction, YAP localization	Direct within defined material models; invasion can increase or decrease with stiffness depending on architecture
Confinement	Nuclear deformation, mode switching, envelope rupture and DNA damage	Width, height, cross-section, length, nuclear-to-pore ratio	Geometry-specific evidence; may increase reliance on contractility, adhesion, proteolysis or nuclear softening
Interstitial flow	Directional bias and chemokine transport	Flow vector and speed, cell trajectory	Direct directional cue; assignment to filament polarity is an inference unless polarity is measured
Solid stress	Vessel compression, hypoxia and spatial heterogeneity	Tissue stress proxies, perfusion, oxygenation	Direct vascular effects; links to active-force organization or defects are hypotheses
Viscoelasticity	Time-dependent force transmission and adhesion turnover	Relaxation time, creep, loading rate	Direct material property; biological outcome depends on timescale matching
ECM degradation	Path opening, ligand exposure and matrikine signaling	Proteolysis, pore evolution, invadopodia	Direct remodeling measurement; downstream actin-state assignment is context-dependent

### 4.1 Mechanical cues and measurable consequences

No absolute confinement cutoff or universal active-force direction is assigned. For confined-migration experiments, complete geometry and nuclear dimensions should replace labels such as “narrow” or “less than 7 μm”.

TACS-3 — collagen fibers oriented perpendicular to the tumor boundary—is associated with poor outcome and was an independent prognostic indicator in a retrospective human breast-cancer cohort (Provenzano et al., 2006; Conklin et al., 2011). TACS-3 is an anisotropic stromal feature that can provide contact guidance. Whether it increases $S_{\text{cell}}$ or predicts tissue-scale defect orientation must be established by paired measurements rather than inferred from collagen alignment alone. LOX-mediated cross-linking stiffens stroma and enhances integrin-dependent mechanotransduction (Levental et al., 2009). Collagen alignment, rather than stiffness alone, supplies the contact-guidance cue for oriented invasion. A clean dissection shows these cues are separable: collagen *bundling* raises invasion independent of migration mode, whereas AGE-mediated *stiffening* selectively blocks collective invasion while sparing single-cell invasion, with collective invasion depending on tumor-cell-derived LOXL3 (Koorman et al., 2022). This gives a microenvironmental version of the same argument: bundling and stiffness are distinct inputs with different effects on invasion, and “stiffness” alone is too coarse a variable.

Solid stress—pressure from growing tumor tissue—compresses vessels (hypoxia) and lymphatics (elevated interstitial pressure) and imposes spatial activity gradients that distort the defect landscape beyond what uniform-activity theory predicts and create spatially variable force-generating and adhesive states, plausibly underlying the phenotypic heterogeneity seen at invasion fronts (Box 2’s “activity gradient” caveat made concrete).

### 4.2 CAF-led invasion and macrophage-assisted intravasation: external force generation

Cancer-associated fibroblasts are active mechanical architects. Heterotypic E-cadherin/N-cadherin contacts let CAFs generate physical traction that pulls poorly invasive cells through dense stroma (Labernadie et al., 2017), with an E-cadherin/DDR1/Par3–Par6 complex limiting actomyosin contractility at these contacts to maintain cohesion (Hidalgo-Carcedo et al., 2011). CAFs deposit Thy1- and MFGE8-containing tracks that steer cancer-cell migration through adhesion involving clathrin-coated structures (Baschieri et al., 2023), and TGF-β triggers reversible single-cell motility (Giampieri et al., 2009). The mechanical behavior of a cancer cell is therefore not cell-autonomous: CAFs can alter the forces, adhesion and local cell-orientation field experienced by neighboring cancer cells. The same non-autonomy operates within the tumor cell population through leader–follower organization: in collective invasion only a subset of cells become protrusive leaders, a specification driven by aligned collagen and a YAP-centered force loop rather than by transcriptional identity alone (Khalil et al., 2024). Consistent with traction-force studies of collective cell migration (Trepat et al., 2009), leader cells generate protrusion and local traction at the strand tip, whereas followers maintain junctional force transmission. Formal high P cannot be assigned without resolving filament polarity. A single strand can therefore contain spatial gradients of force transmission, protrusion and cell-body alignment rather than one uniform mechanical state.

Tumor-associated macrophages participate in the CSF-1/EGF paracrine loop first resolved by intravital imaging approaches from the Condeelis/Segall lineage (Condeelis and Segall, 2003; Wyckoff et al., 2004)—carcinoma cells secrete CSF-1, recruiting macrophages that secrete EGF, stimulating tumor-cell protrusion and co-migration toward vessels. Assignment to P would require polarity-resolved actin data. The TMEM (tumor microenvironment of metastasis) score—tripartite tumor-cell/macrophage/endothelial structures—predicts distant-metastatic risk in ER^+^/HER2^-^ breast cancer (Rohan et al., 2014). TAM-mediated invasion shows the cancer-cell mechanical state cannot be understood in isolation from stromal and immune activity—a concrete instance of the multi-species limitation single-species active-nematic models face (Box 2).

### 4.3 Actin mechanics at the tumor–immune interface

Dense, aligned collagen can physically impede T-cell penetration: real-time imaging in human lung-tumor slices shows T cells migrate freely in loose matrix but poorly in dense regions, with fibers aligned around tumor-cell nests channeling them away and restricting entry (Salmon et al., 2012). Reducing matrix stiffness by inhibiting collagen crosslinking increases intratumoral T-cell migration and improves anti-PD-1 response, linking the mechanical state of the stroma to immune accessibility (Nicolas-Boluda et al., 2021); matrix stiffness has also been linked to M2-like macrophage accumulation (Taufalele et al., 2023), plausibly building immunosuppressive niches, and tumor-cell actomyosin contractility may remodel the secretome in ways that exclude T cells (§3.1, the JAK1/STAT3–ROCK loop). These connections have not been tested as a single mechanical-state-to-immune-accessibility axis. Whether cortical force organization, $S_{\text{ECM}}$ , stiffness and solid stress predict immune accessibility or immunotherapy response is an important unresolved question: if even partly true, it connects the mechanical measurements to a clinically consequential question. We return to it as a testable gap (§7).

## 5 Cancer ecologies: what is established and what is extrapolated

**FIGURE 4:**
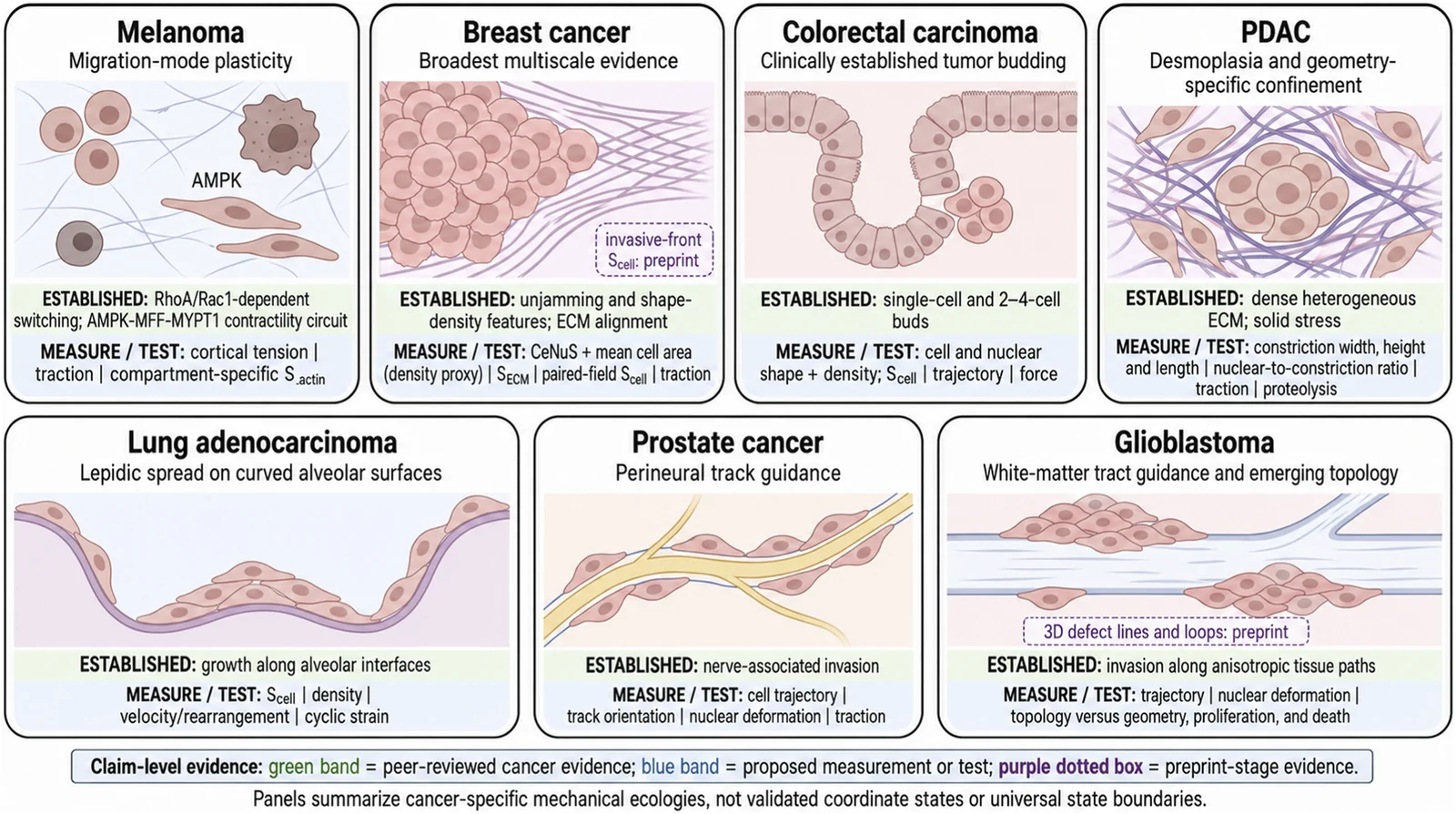
Cancer-specific mechanical ecologies: established evidence and testable measurements. The panels compare melanoma, breast cancer, colorectal carcinoma, pancreatic ductal adenocarcinoma, lung adenocarcinoma, prostate cancer and glioblastoma without assigning any cancer a fixed mechanical state. Green bands identify peer-reviewed cancer evidence for the named claim; blue bands identify proposed measurements or tests; and purple dotted boxes identify preprint-stage evidence. The breast-cancer panel separates established unjamming, shape-density features and ECM alignment from preprint-stage invasive-front cell alignment. Colorectal tumor budding is clinically established, whereas its mechanical signature remains to be measured. The remaining panels likewise distinguish established ecology from candidate measurements. These are claim-level evidence categories, not validated coordinate states or universal state boundaries.

The evidence is not distributed evenly across tumor types. Melanoma provides the clearest molecular studies of rounded-versus-elongated switching. Breast cancer supplies the strongest integration of three-dimensional invasion, patient-derived fluidity, stromal alignment and prognosis. Colorectal tumor budding offers a standardized pathology endpoint. The remaining examples are used to define boundary conditions, not to imply that the same mechanical classification has already been demonstrated in each disease. Table 2 records the strongest evidence level and leaves absent measurements blank; Figure 4 provides the corresponding visual comparison.

**TABLE 2:** Evidence and model-system matrix.

Evidence layer	Melanoma	Breast	Cervix/explants	CRC	Airway/lung model
A) Better-characterized cancer and epithelial systems					
Active-force switching	Mapped	—	—	—	—
Shape-density/unjamming	—	DCIS; patient cohort	Patient explants	Budding testable	Bronchial model*
Tissue defects	Dermal flow only; no defects mapped	Invasive front (preprint)	—	—	—
AMPK-contractility	MFF–MYPT1	—	—	—	—
Invadopodia	Cortactin–cofilin	Cortactin–cofilin	—	—	—
YAP/TAZ loop	—	Collagen–YAP	—	—	—
Stiffness/rigidity	—	LOX–collagen; soft cells in rigid tumors	Soft cells in rigid tumors	—	—
Budding	—	—	—	ITBCC	—
Paracrine	—	CSF-1/EGF/TMEM	—	—	—

Evidence layer	PDAC	GBM	Prostate	MDCK	U2OS/screens
B) Sparse, hypothesized or model-system layers					
Active-force switching	—	—	—	—	—
Shape-density/unjamming	Hypothesized under desmoplasia	—	—	Established non-cancer epithelial model	—
Tissue defects	—	3D *in vivo* organization (preprint)	—	Established non-cancer epithelial model	—
AMPK-contractility	—	—	SNPH axis	—	—
Invadopodia	—	—	—	—	—
YAP/TAZ loop	Hypothesized via stiffness	—	—	—	—
Stiffness/rigidity	Desmoplastic extreme	EGFR–stiffness	—	—	—
Screens	—	—	—	—	JUMP cell painting; CRISPRi

Entries indicate the strongest evidence class for each relationship. “Preprint” means not yet journal peer-reviewed; “bronchial” and “MDCK” are non-cancer epithelial models; U2OS entries are high-content feature-discovery resources rather than invasion models. Em dashes indicate that relevant evidence was not identified for the stated system; they do not demonstrate biological absence.

### 5.1 Melanoma: strong evidence for migration-mode switching

Melanoma remains the best-characterized system for RhoA/Rac1-dependent interconversion between elongated and rounded invasion (Sanz-Moreno et al., 2008; Sanz-Moreno et al., 2011). The AMPK-MFF-MYPT1 circuit links energetic stress, mitochondrial fission and sustained actomyosin contractility (Crosas-Molist et al., 2023), while bleb-driven “worrying” provides a proteolysis-independent route through collagen (Driscoll et al., 2024). These findings support a force-organization description because the underlying regulators, morphology and invasion behavior were studied together.

The limitation is scope. Melanoma is non-epithelial, usually lacks the confluent junctional architecture assumed by vertex and epithelial-nematic models, and is therefore a poor system from which to infer universal shape-density or defect rules. Even within melanoma, rounded and elongated categories compress continuous, spatially heterogeneous behavior. The literature establishes plasticity between migration programs; it does not validate one scalar active-stress coordinate.

### 5.2 Breast cancer: the broadest evidence, with unresolved coupling

Breast cancer provides the most complete test bed. RAB5A-driven MCF10A.DCIS collectives unjam while retaining epithelial features (Palamidessi et al., 2019). Three-dimensional breast-cancer invasion depends jointly on cell-cell adhesion and available extracellular space (Ilina et al., 2020). Patient studies relate cell-and-nuclear shape and density to motility and distant metastasis (Gottheil et al., 2023), while collagen alignment, YAP-centered mechanotransduction and TMEM structures connect stromal mechanics to invasion and prognosis (Provenzano et al., 2006; Rohan et al., 2014; Khalil et al., 2024).

These observations do not converge on a single mechanical trajectory. E-cadherin loss can facilitate individualization under permissive geometry, yet E-cadherin is required for metastasis in several breast-cancer models (Padmanaban et al., 2019). Collagen bundling and stiffness can have separable effects on collective and single-cell invasion (Koorman et al., 2022). Breast cancer therefore demonstrates the conditional nature of these relationships: adhesion, confinement, stromal alignment and transcriptional state must be measured together. Reports of tumor-cell nematic alignment at invasive fronts are potentially important but remain preprint-stage (Gottheil et al., 2024) and should not yet carry the same evidentiary weight as the patient shape-density studies.

The principal missing bridge is time-resolved subcellular organization. Static phalloidin texture, cell shape, migration speed and tissue $S_{\text{cell}}$ do not establish how actin-associated motion is organized within a living cancer cell. Flow-derived $S_{\text{flow}}$ , polar coherence and persistence could fill this gap, but an initial comparison across a small set of established cell lines would demonstrate cell-state separability, not a general metastatic law. The required extension is perturbational and hierarchical: additional independently derived models, three-dimensional or organoid imaging, direct force readouts and patient-linked validation.

### 5.3 Colorectal carcinoma and tumor budding—the most practical pathology entry point

We foreground colorectal carcinoma because tumor budding makes the proposal easy to test with existing material and standardized scoring, not because it is a neutral dissociation case. Budding—a single cell or cluster of up to four cells detaching at the invasive front—is a clinically established prognostic marker formalized by the International Tumor Budding Consensus Conference (Lugli et al., 2017); high budding predicts lymph-node metastasis, recurrence and cancer-related death in meta-analysis (Rogers et al., 2016). It is attractive because it occurs at the invasion front, reflects epithelial plasticity, and is already quantified by a protocol embedded in pathology practice.

The conceptual tension should be explicit. Budding has been linked to partial-EMT markers—loss of membrane E-cadherin, nuclear β-catenin, ZEB1/2 — and has even been argued to be partial EMT by definition (Grigore et al., 2016). That makes budding a direct comparison between transcriptional and mechanical interpretations rather than an unbiased test. The hypothesis is therefore not that budding lacks EMT features. It is that budding regions may carry additional mechanical information in q , context-dependent $S_{\text{cell}}$ , active-force proxies or defect geometry—whose explanatory or prognostic value can be tested after measuring EMT markers in the same regions. The translational question is concrete: can ITBCC scores be improved by adding quantitative shape (*q*) and cell-orientation ( $S_{\text{cell}}$ ) features extracted from the same section, where boundaries are resolvable or membrane-assisted segmentation is available? The principal dissociation test remains the matched spatial-omics/multiplexed-imaging experiment in §7; CRC budding is the lowest-burden pathology screen that can be run first.

### 5.4 Pancreatic ductal adenocarcinoma: a test of environmental dominance

PDAC is dominated by desmoplasia, solid stress, irregular pore geometry and immune exclusion. Those features make stromal material properties and nuclear-to-pore geometry more immediately relevant than a cell-autonomous state label. Aligned collagen may provide contact guidance, while dense matrix can increase reliance on proteolysis, adhesion-based traction, contractility or nuclear softening. Which route is used cannot be inferred from desmoplasia alone. Because gland boundaries are difficult to segment and two-dimensional cell perimeter is highly section-dependent, a universal q cutoff would be especially poorly justified in PDAC. The disease is a valuable test of whether the proposed measurements generalize, but direct multiscale evidence remains sparse.

### 5.5 Lung adenocarcinoma: geometry may permit, but does not prove, a two-dimensional analogy

Lepidic spread along alveolar surfaces resembles a quasi-two-dimensional collective and may be experimentally amenable to orientation and fluidity analysis. The analogy is incomplete: the alveolar interface is curved and discontinuous, basement membrane is thin, surfactant alters adhesion, and cyclic stretch continually perturbs force transmission. Once cells breach the basement membrane, three-dimensional matrix geometry and nuclear deformability become dominant. Lung adenocarcinoma should therefore be used to test where a two-dimensional description fails, not described as an already established active nematic.

### 5.6 Prostate cancer and perineural invasion: anisotropic tracks are not a force state

Perineural invasion supplies a strongly anisotropic path, but directional guidance by a nerve does not by itself establish high actin order or high cortical contractility. Rapid perineural invasion can use amoeboid migration in pancreatic cancer (Marcadis et al., 2023), but the dominant force-transmission mechanism in prostate patient tissue has not been established. The relevant measurements are track geometry, cell and nuclear deformation, adhesion engagement and force organization. The SNPH-mitochondrial axis is mechanistically interesting (Caino et al., 2017), but it has not been integrated with these measurements in patient tissue.

### 5.7 Glioblastoma: emerging topology, preliminary tumor evidence

Glioblastoma cells exploit white-matter tracts and perivascular spaces, so substrate anisotropy, elongation and nuclear mechanics are more relevant than epithelial shape-density models. Alpha-actinin isoforms contribute to rigidity sensing and motility (Sen et al., 2009), whereas substrate stiffness can amplify EGFR signaling and proliferation (Umesh et al., 2014), and aligned multicellular oncostreams have been described (Comba et al., 2022). A recent preprint reports three-dimensional nematic order and disclination structures in glioma, with defect class associated with different apoptotic behavior (Argento et al., 2025).

If replicated, that study would materially strengthen the tumor relevance of three-dimensional defect topology. At present it remains insufficient to claim that defects organize human glioblastoma invasion or predict outcome. The critical controls are geometry, proliferation, death and sectioning: each can generate or alter apparent defects independently of a causal active-nematic mechanism.

### 5.8 Epithelial-mesenchymal plasticity and mechanics: coupled, not interchangeable

The literature supports neither a mechanics-only nor a transcription-only account of invasion. Unjamming with retained epithelial markers demonstrates that collective motility can emerge without a canonical EMT program in airway epithelium and selected carcinoma models (Mitchel et al., 2020; Palamidessi et al., 2019). Patient-derived breast and cervix tissues contain shape-density states associated with motility and metastatic outcome (Grosser et al., 2021; Gottheil et al., 2023). These results establish that mechanical morphology deserves measurement in its own right.

The opposing evidence is equally important. In breast-cancer models, E-cadherin can be required for collective dissemination and metastasis (Padmanaban et al., 2019), whereas loss or weakening of cadherin complexes can facilitate stepwise individualization in a confinement-dependent manner (Ilina et al., 2020). EMT may be dispensable for formation of some lung metastases yet contribute materially to chemoresistance (Fischer et al., 2015). EMT regulators also act through mechanically relevant effector programs: the ZEB1-miR-200c axis controls the invadopodial scaffold Tks5/SH3PXD2A and MYLK (Sundararajan et al., 2015), while TWIST1 can promote invadopodia through PDGFRα-Src signaling (Eckert et al., 2011). These links argue against treating transcriptional and mechanical state as independent layers. Moreover, patient unjamming studies have not systematically measured EMT-transcription-factor activity and live mechanics in the same cells. Consequently, the available data show separability in specific systems, coupling in others, and no basis for a universal one-to-one mapping.

Table 3 therefore replaces categorical phenotype assignments with compartment- and context-specific patterns. The entries are expectations to be tested, not state boundaries.

**TABLE 3:** Context-dependent mechanical patterns across invasion phenotypes.

Phenotype or region	Actin organization	Cell/tissue morphology	Force and adhesion interpretation
Jammed cobblestone epithelium	Cortical network; stress fibers variable	Low elongation; $S_{\text{cell}}$ often low or poorly defined	Junctionally coupled; low rearrangement rate, but cortical tension may be high
Unjamming epithelial collective	Protrusive leader edge; formal P requires polarity-resolved actin data; follower organization heterogeneous	Increased elongation and alignment in many systems; density and speed must be reported	Propulsion, junctional remodeling and confinement contribute; EMT may be absent or coupled
Mesenchymal single cell	High leading-edge polarity; low $S_{\text{actin}}^{LE}$ can coexist with high $S_{\text{actin}}^{SF}$	Elongated shape is common but not diagnostic	Adhesion-dependent traction and proteolysis are frequent, not universal
Rounded/amoeboid cell	Cortex may be globally isotropic despite local arcs or leader-bleb organization	Rounded shape; $S_{\text{cell}}$ weakly defined	High isotropic cortical contractility can coexist with low substrate traction
Tumor bud	Compartmental actin data sparse	One to four cells by ITBCC in CRC; shape depends on section	Mixed adhesion and force states; no validated single mechanical signature

The table deliberately avoids “high” or “low” whole-cell nematic order where subcellular compartments can have opposing values. Cell-body order is not used as an epithelial marker. No $S_{\text{flow}}$ value is assigned from static morphology; it requires live image analysis and an explicitly defined velocity estimator.

## 6 Controversies and boundary conditions

### 6.1 Is unjamming independent of EMT?

The strongest answer is conditional. In primary bronchial epithelium, unjamming and EMT can be experimentally separated, with different changes in propulsion and junctional tension (Mitchel et al., 2020). RAB5A-driven collective motility in a breast carcinoma model also preserves epithelial features (Palamidessi et al., 2019). These studies disprove the universal claim that motility requires complete EMT.

They do not prove that unjamming is transcriptionally independent in human cancers. Tumors contain hybrid EMT states, altered cadherin expression and strong stromal constraints. Ilina et al. (2020) showed that cell-cell adhesion and confinement jointly govern coordinated motion and individualization. Padmanaban et al. (2019) showed that E-cadherin can be required for metastasis in breast-cancer models. Resolution requires same-cell measurement of live fluidity and EMT/EMP activity, not comparison of separate cohorts or systems.

### 6.2 Does more contractility mean more invasion?

No monotonic rule is defensible. Increased cortical contractility can support blebbing, squeezing and survival under mechanical stress. Excessive contractility can also reduce protrusive persistence, disrupt productive adhesion or impair collective coordination. MYH9 can oppose squamous-cell tumorigenesis through p53-related effects (Schramek et al., 2014). The relevant variables are the magnitude, spatial organization and transmission of force. A study reporting only pMLC, only traction or only cell shape cannot distinguish them.

### 6.3 Are active-nematic defects causes or consequences?

Defects can organize stress and extrusion in controlled monolayers (Saw et al., 2017), establishing that topology can be mechanistically consequential. In tumors, however, defects can also arise passively from boundaries, crowding, proliferation, cell death or imposed collagen alignment. A correlation between defect density and invasion would not distinguish cause from consequence. Causality requires perturbing orientation or defect position while controlling geometry and measuring the predicted change in invasion or extrusion.

### 6.4 Can static histology infer dynamics?

Static morphology can predict dynamic state only after calibration. The Gottheil et al. (2023) framework is notable because shape-density features from histology were related to motility in living tumor explants. That logic should not be generalized to arbitrary cell shape indices. Routine H&E resolves nuclei, cytoplasm and tissue architecture but usually not complete cell membranes. Nuclear-guided watershed or inferred boundaries impose a model on perimeter and therefore on q . Two-dimensional sections further distort three-dimensional area, perimeter and orientation according to section depth and cutting angle.

True cell-boundary q requires membrane-resolved staining or a segmentation method validated against such staining. Studies should report boundary uncertainty, inter-annotator and inter-pipeline reproducibility, sensitivity to section thickness and orientation, and uncertainty propagation into the final classifier. A claimed association with q should survive plausible boundary perturbations, alternative segmentation pipelines and analysis of a membrane-ground-truth subset. In routine H&E, nuclear shape, gland architecture, CeNuS-like features and density are more defensible than an unqualified single-cell q .

### 6.5 Are the proposed observables independent?

Usually not. Shape and cell-body orientation share a segmentation; defects are derived from that orientation field; anisotropic active stress contains the nematic tensor; and several actin metrics share the same images. The scientific objective is not to force independence but to establish identifiability. Multimodal analyses should use preregistered definitions, measurement-error models, covariance analysis, ablation of redundant features and out-of-sample validation. Incremental clinical value must be assessed after grade, stage, subtype, density and EMT/EMP state are included.

### 6.6 Can a molecular module stand in for a measured mechanical state?

No. A transcriptional or proteomic module can identify molecular machinery that is compatible with a mechanical phenotype, but it does not measure that phenotype. This distinction is especially important for modules selected partly by differential expression between cell lines or disease groups: separation of those same groups by the resulting score is expected by design and is not independent validation. Dependency data, protein detectability, network connectivity, invasive-front enrichment and outcome associations can strengthen the biological plausibility of a module, but none calibrates $S_{\text{flow}}$ , cortical tension or traction in the cells from which the omics measurement was obtained.

The required bridge is paired measurement. Live actin-associated motion, direct cytoskeletal architecture, force transmission and molecular state should be measured in the same perturbed cells or matched organoids. A mechanically annotated molecular score should then predict the held-out physical measurement after cell lineage, subtype and EMT/EMP are controlled. Until that test is met, terms such as “nematic-associated module” are defensible, whereas “nematic program” should be understood as shorthand for association rather than identity.

### 6.7 Our position

The evidence supports a multiscale mechanical *description* of invasion, but not a universal mechanical phase diagram for cancer. The most mature route to translation is a calibrated, multivariate shape-density classifier combined with molecular and histopathological covariates. The least mature route is direct transfer of defect rules or active-stress signs from quasi-two-dimensional monolayers to heterogeneous three-dimensional tumors. Between these extremes, dynamic actin-flow order is a promising subcellular descriptor because it is measurable in living cells and yields explicit temporal predictions. Its interpretation remains narrower than structural filament order, active stress or metastatic competence until those links are tested directly. This evidentiary ranking specifies where current knowledge ends.

## 7 Measurement and validation strategy

**FIGURE 5:**
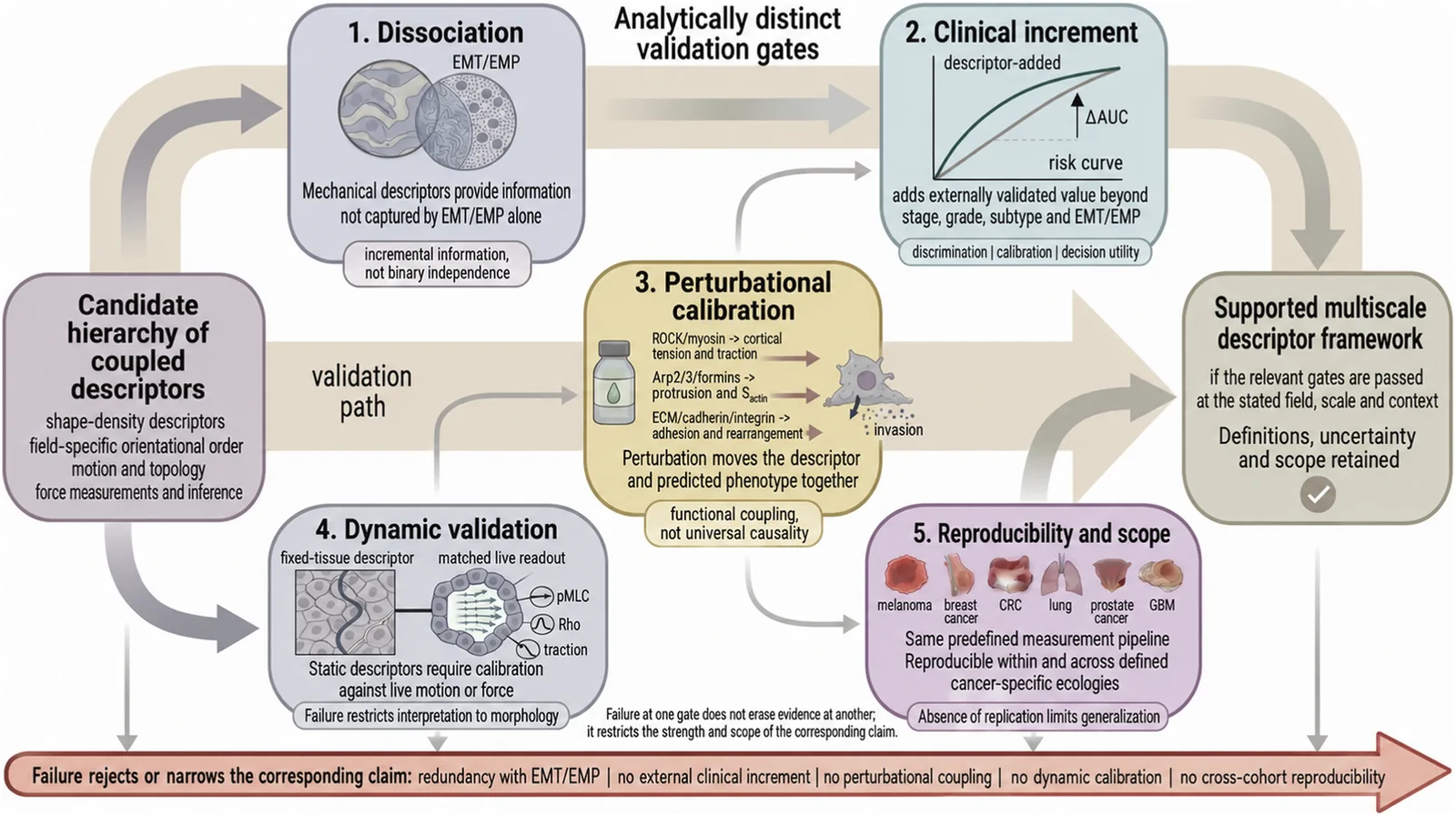
Analytically distinct validation gates and claim-level failure criteria. A candidate hierarchy of coupled descriptors is evaluated through five analytically distinct gates: incremental information beyond EMT/EMP; externally validated clinical increment beyond established covariates; perturbational coupling between a descriptor and the predicted phenotype; calibration of static descriptors against live motion or force; and reproducibility within and across defined cancer-specific ecologies using the same prespecified pipeline. Passing the relevant gates supports a multiscale descriptor framework only at the stated field, scale and context. Failure at one gate does not erase evidence established at another, but it rejects or narrows the corresponding mechanistic, dynamic, clinical or generalization claim.

**FIGURE 6:**
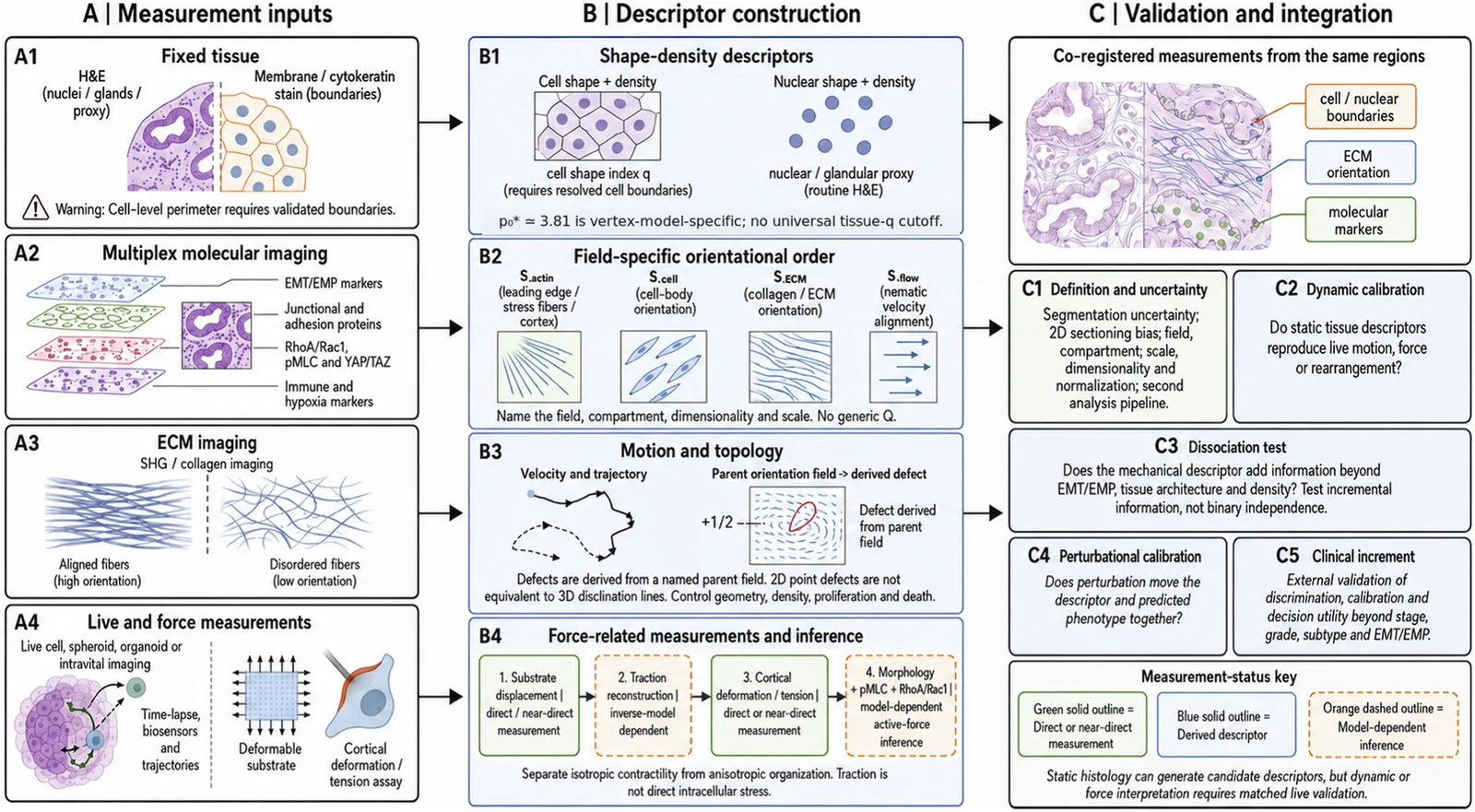
From tumor measurements to validated multiscale descriptors. **(A)** Fixed-tissue morphology, multiplex molecular imaging, ECM imaging and live or force measurements provide distinct inputs. Routine H&E primarily supports nuclear, glandular or inferred-cell proxies; cell-level shape index requires validated membrane or cytokeratin-resolved boundaries. **(B)** Shape-density descriptors, field-specific orientational order, motion, topology and force-related readouts are constructed with explicit field, compartment, dimensionality and uncertainty. The canonical vertex-model target-shape value is model specific, not a universal tissue cutoff. Substrate displacement and cortical deformation are direct or near-direct observations; traction reconstruction and active-force classification are model dependent; and defects are derived from a named parent field. **(C)** Co-registered measurements are evaluated through definition and uncertainty, dynamic calibration, incremental information beyond EMT/EMP and architecture, perturbational calibration and external clinical validation. Static histology cannot establish dynamic or force interpretation without matched live validation.

Figure 5 summarizes the proposed validation roadmap, and Figure 6 distinguishes measurements from image-derived or model-dependent estimates. The validation gates are analytically distinct: failure at one gate restricts the corresponding claim and prevents escalation to a stronger evidence level, but does not erase evidence established at another gate.

### 7.1 Measurement tiers

Direct or near-direct physical measurements include cortical deformation or tension assays, substrate displacements and tracked cell/nuclear trajectories. RhoA/Rac1 biosensors and pMLC are direct molecular-activity measurements used for mechanistic triangulation; they are not mechanical readouts. Image-derived dynamic estimates include optical-flow fields and the $S_{\text{flow}}$ , $P_{\text{flow}}$ , strain, divergence and vorticity calculated from them. Static derived descriptors include q , compartment-specific structural S , density and adhesion morphology. Model-dependent inferences include intracellular active-stress parameters, an unjamming-state classifier transferred to a new cohort, and the biological meaning of a defect map. These tiers should not be pooled under the label “measured.”

### 7.2 Minimum reporting standards

For orientation analysis, authors should report the imaged field, spatial window, smoothing, dimensionality, normalization and confidence of the principal axis; near-isotropic cells should be excluded or confidence-weighted in sensitivity analyses. For optical flow, the algorithm, regularization, brightness preprocessing, pixel size, frame interval, mask, weighting, confidence definition and parameter-sensitivity range must be disclosed. Polar coherence and apolar alignment should be reported together when the underlying field is a velocity. Agreement with a second flow estimator tests algorithmic robustness, whereas comparison with filament-resolved orientation or speckle/particle tracking tests biological validity. Temporal statistics must use cells or biological replicates, not frames, as independent units. Defect claims require a named parent field, the exact phase-winding convention and stability across justified smoothing scales and boundary treatments. For confinement, complete geometry and nuclear dimensions are required. For TFM, the substrate model, elastic or viscoelastic parameters, boundary conditions, regularization and propagated uncertainty must be specified. For histological shape analysis, staining, section thickness, membrane ground truth, segmentation algorithm, boundary perturbation, failure cases and sensitivity to sectioning must be disclosed.

### 7.3 Tests with the greatest information gain

The highest-priority subcellular test is same-cell validation of dynamic actin-flow order. $S_{\text{flow}}$ and $P_{\text{flow}}$ should be compared with filament-resolved or structure-tensor actin orientation, migration direction, traction or cortical tension, and a functional invasion endpoint during controlled perturbation. This design can determine whether flow order reports structural organization, force production, transport coherence or a combination of these. A static filament image failing to reproduce dynamic flow order would establish non-equivalence rather than invalidate the dynamic measurement.

A second priority is a same-specimen study combining live motility or organoid rheology, membrane-resolved morphology and spatial EMT/EMP markers. Its purpose is to determine whether a mechanical descriptor adds information after transcriptional state, density and tumor architecture are controlled. The appropriate outputs are partial associations, calibrated prediction, external validation and uncertainty—not a *post hoc* threshold.

A third priority is mechanistic validation of active-force organization. RhoA/Rac1 biosensors, cortical tension, pMLC distribution, stress-fiber order, focal-adhesion engagement and TFM should be measured together during mode switching. This design can determine which measurements distinguish isotropic cortical contractility from anisotropic stress and substrate traction.

A fourth priority is causal testing of tissue topology. Defect position or orientation should be perturbed without changing density or boundary geometry, followed by prospective measurement of extrusion or invasion. Until such experiments succeed in tumors, defect maps remain descriptive.

High-content perturbation provides a complementary discovery route. Genome-wide RNAi morphology screening has identified regulators of cytoskeletal organization and migration (Bai et al., 2011), while the JUMP Cell Painting resource enables systematic chemical and genetic feature discovery (Chandrasekaran et al., 2024). These resources are not themselves invasion models. Candidate features must be transferred into relevant cancer systems and tested against direct structural, force and motility measurements before mechanistic interpretation.

Circulation supplies a distinct mechanical selection regime. Blood-flow forces and endothelial adhesion govern arrest and extravasation in zebrafish models (Follain et al., 2018), and human brain metastases preferentially occur in defined perfusion and anatomical regions (Barrios et al., 2025). CTC clusters have markedly increased metastatic potential and preserve plakoglobin-dependent cohesion during dissemination (Aceto et al., 2014), while RhoA-actomyosin-dependent shear resistance can promote circulating-cell survival (Moose et al., 2020). These observations justify studying collective order and contractility in circulation, but neither CTC clustering nor shear survival establishes the active-stress tensor of a primary tumor.

### 7.4 Prospective test matrix

Table 4 makes the experimental commitments explicit. It also separates feasible pathology studies from mechanistic force validation and longer-term three-dimensional topology experiments. Failure criteria are stated prospectively so that negative findings cannot be absorbed by redefining a state after inspection.

**TABLE 4:** Testable predictions and interpretation criteria, ranked by near-term feasibility. Numerical criteria are prospective design examples to be fixed before analysis; they are not validated cancer-state boundaries.

Question	Prospective test	Evidence that supports or weakens the claim	Feasibility
Does a shape-density descriptor add information beyond EMT/EMP?	Membrane-resolved morphology, density and spatial EMT/EMP measurements in the same invasive-front and internal regions, with tumor-grouped validation	Supports: reproducible incremental discrimination or calibration in an external cohort. Weakens: no increment after architecture, density and EMT/EMP are controlled	Near-term
Is CRC budding associated with altered cell shape?	ITBCC-scored budding regions with membrane/cytokeratin-resolved boundaries; compare q with matched non-budding front regions	Supports: pre-specified effect reproduced across cohorts. Weakens: effect disappears after boundary uncertainty and sectioning are modeled	Immediate to near-term
Does a shape-density classifier add prognostic value?	Cox or competing-risk model including stage, grade, subtype and molecular covariates; external validation and calibration	Supports: independent, reproducible clinical increment. Weakens: no value beyond standard pathology	Near-term
Does actin-flow order report filament organization or force?	Same-cell $S_{\text{flow}}$ , $P_{\text{flow}}$ , filament-resolved orientation, traction/cortical tension and invasion during perturbation	Supports: pre-specified coupling that survives algorithm and replicate sensitivity analyses. Weakens: classifier is algorithm-specific or unrelated to direct structure/force	Near- to mid-term
Can active-force organization be classified from images?	Paired morphology, RhoA/Rac1, pMLC, cortical tension and TFM in the same cells	Supports: externally validated distinction between isotropic cortical contractility, anisotropic organization and substrate traction. Weakens: image features fail matched-force validation	Mid-term
Does force organization predict perturbation response?	Pre-profiled cells challenged with ROCK/myosin, Arp2/3 or adhesion perturbations, with mode switching and viability measured	Supports: differential response predicted prospectively. Weakens: response is unrelated to baseline force organization	Mid-term
Does YAP localization track a defined mechanical variable?	Matched YAP localization, cortical tension, traction and density measurements	Supports: association remains after density and cell-cycle adjustment. Weakens: association is explained by confounding variables	Mid-term
Does AMPK-associated contractility generalize beyond melanoma?	AMPK, MFF, MYPT1 and direct force readouts in independent PDAC, CRC, breast and lung models	Supports: replicated coupling in multiple independently derived systems. Weakens: melanoma-restricted association	Multi-year
Does compartment-specific structural actin order predict migration mode?	Leading-edge, stress-fiber and cortical $S_{\text{actin}}$ measured before lamellipodial or bleb transitions, controlling confinement, adhesion and pMLC	Supports: prospective compartment-specific relationship. Weakens: whole-cell or compartmental order adds no predictive information	Mid-term
Do +1/2 defects prospectively identify invasion sites?	Live organoid surface mapping or true three-dimensional disclination-line analysis, with randomized-field and matched-geometry controls	Supports: pre-specified spatial enrichment followed by causal perturbation. Weakens: enrichment vanishes after geometry, density and proliferation are controlled	Far-term
Does Arp2/3 inhibition preserve protrusive polarity through formin compensation?	Polarity-resolved actin imaging after CK-666 with and without FMNL2/3 perturbation	Supports: P is preserved after Arp2/3 inhibition but collapses with combined formin perturbation. Weakens: P collapses with Arp2/3 inhibition alone	Mid-term

### 7.5 Translational claims and falsification criteria

Mechanical features should be evaluated against, not instead of, standard pathology. A proposed classifier is clinically informative only if it improves discrimination, calibration or decision utility beyond stage, grade, subtype and relevant molecular markers in an external cohort. A feature that is reproducible but redundant may still illuminate mechanism, but it should not be presented as a new biomarker.

Cell-line classification is an earlier evidentiary stage. Correctly separating cells from different lines demonstrates phenotypic information, not prediction of metastatic behavior, because lineage, subtype and genotype are inseparable from the class label. Frames are repeated observations within cells and cells are nested within lines; neither should be treated as independent replication of a metastatic category. Claims of a metastasis-associated gradient require additional independent cell lines, patient-derived models and prospective invasion or dissemination endpoints, with cross-validation grouped at the biological-replicate or model level.

A universal version of the proposal would be rejected if shape-density or force features add no information after EMT/EMP and architecture are modeled, if static proxies fail prospective dynamic validation, or if defect associations vanish after controlling geometry and proliferation. Conversely, success in one tumor type would not establish universality; cross-cancer generalization requires independent calibration because tissue architecture and invasion ecology differ.

## 8 Discussion and outlook

Cancer invasion necessarily involves mechanical processes, but that fact alone does not make every mechanical quantity an independent coordinate or a clinical biomarker. The literature currently supports a hierarchy of coupled observables. Filament and cell orientations, force-related measurements, boundaries and image sequences are primary data; polarity, structural nematic order, flow-derived alignment, shape and density are derived descriptors; defects and unjamming states are higher-order constructions. Their value depends on scale, compartment, geometry and inference model.

The synthesis supports three refinements to the central proposition. First, isotropic cortical contractility is separated from anisotropic active stress and substrate traction. Second, the vertex-model parameter $p_{0}^{*}≃3.81$ is separated from observed tumor-cell shape and from the multivariate CeNuS-density state diagram used in patient tissue. Third, the proposed observables are treated as dependent measurements whose incremental information must be demonstrated. Structural actin order, apolar flow alignment and polar flow coherence are also separated, preventing a dynamic image statistic from being misidentified as filament architecture or stress.

The evidence for mechanical-transcriptional dissociation is important but bounded. Unjamming can occur without canonical EMT in defined epithelia and carcinoma models, yet cadherin regulation, EMT/EMP and confinement can also cooperate in invasion and metastasis. Contractility can promote or restrain malignancy, depending on its organization and molecular context. Defects can be causal organizers in monolayers or passive consequences of tumor geometry. Static histology can serve as a dynamic proxy only after explicit calibration. Likewise, a molecular score can be associated with a mechanical state without measuring it; paired same-cell validation is required to cross that evidentiary boundary.

The most credible near-term advance is therefore not a universal mechanical phase diagram. It is a multimodal measurement program that asks whether well-defined mechanical observables improve explanation or prediction after established molecular and pathological variables are considered. That question is demanding, falsifiable and clinically relevant. Answering it would establish where mechanics contributes genuinely new information—and where it is simply another view of the same underlying state.

BOX 1. **Formal definitions and evidence status**

Evidence labels used in the figures and tables are: direct measurement, derived descriptor, model-dependent inference, cancer-model validation and patient-cohort validation. A numerical range is not an experimentally validated cancer-state boundary. A value derived repeatedly from many frames does not increase the number of biological replicates.

**Table:** 

Quantity	Formal definition	Range	Direct measurement or inference
Polar order P	$P=\langle t\cdot m\rangle =\langle \cos\theta \rangle$ , with barbed-end direction t	−1 to 1	Direct only when filament polarity is resolved; flow coherence is a proxy
Two-dimensional tensor $Q_{ij}^{\left(2D\right)}$	$Q_{ij}^{\left(2D\right)}=\langle n_{i}n_{j}-\delta_{ij}/2\rangle$	Eigenvalues −1/2 to 1/2	Derived from a specified two-dimensional orientation field
Normalized two-dimensional order $S_{2D}$	$S_{2D}=2\lambda_{\max}\left(Q^{\left(2D\right)}\right)=\left|\langle e^{2i\theta }\rangle \right|$	0 To 1	Normalized scalar order; source field must be named
Flow alignment $S_{\text{flow}}$	$Q_{ij}^{\text{flow}}=\frac{\langle w\left({\hat{v}}_{i}{\hat{v}}_{j}-\delta_{ij}/2\right)\rangle }{\langle w\rangle }S_{\text{flow}}=2\lambda_{\max}\left(Q^{\text{flow}}\right)$	0 To 1	Derived from an algorithm-estimated velocity field; not filament order or stress
Polar flow coherence $P_{\text{flow}}$	$P_{\text{flow}}=\left|\langle w\hat{v}\rangle /\langle w\rangle \right|$	0 To 1	Retains velocity direction and complements apolar $S_{\text{flow}}$
Three-dimensional tensor $Q_{ij}^{\left(3D\right)}$	$Q_{ij}^{\left(3D\right)}=\langle n_{i}n_{j}-\delta_{ij}/3\rangle$	Traceless tensor	Derived from a specified three-dimensional orientation field
Uniaxial three-dimensional order $S_{3D}$	$S_{3D}=\left(3/2\right)\lambda_{\max}\left(Q^{\left(3D\right)}\right)$	0 To 1	Full eigenvalue spectrum is required for biaxial fields
Active stress $\sigma_{ij}^{\text{active}}$	$\sigma_{ij}^{\text{active}}=-\Pi_{a}\delta_{ij}+\zeta_{a}\Delta \mu Q_{ij}$	Stress units	$\Pi_{a}$ and $\zeta_{a}$ are model parameters; TFM does not measure them directly
Shape index q	$q=P_{\text{cell}}/\sqrt{A_{\text{cell}}}$	$q\geq 2\sqrt{\pi }$ for a planar closed curve	Derived from a validated cell boundary
Vertex target shape $p_{0}$	$p_{0}=P_{0}/\sqrt{A_{0}}$	$p_{0}^{*}≃3.81$ in the canonical confluent two-dimensional model	Model parameter, not a patient-cell cutoff
Defect charge s	$s={\left(2\pi \right)}^{-1}\oint d\theta ={\left(4\pi \right)}^{-1}\oint d\left(2\theta \right)$	Commonly +1/2 or −1/2 for nematics	Derived from a named, smoothed orientation field; not an independent measurement

BOX 2. **Where active-matter and jamming analogies break.**

Active-matter and jamming models have provided rigorous hypotheses for epithelial mechanics. Their transfer to tumors is restricted by six violations of canonical assumptions: tumor cells are polydisperse; division and death continually alter topology; invasion fronts are open boundaries; activity and oxygenation are spatially heterogeneous; the tissue contains multiple interacting cell types; and most tumors are three-dimensional. Point defects in two dimensions become lines or loops in three dimensions, and a two-dimensional section can create or remove apparent singularities.

The $p_{0}^{*}≃3.81$ result belongs to a confluent two-dimensional vertex model. It is useful as a mechanistic benchmark, not a universal experimental threshold. Self-propulsion, persistence, density, heterogeneity and matrix coupling change the transition (Bi et al., 2015; Bi et al., 2016). Similarly, the sign of a tissue-scale active-nematic coefficient cannot be inferred from RhoA activity or whole-cell traction without coarse-graining the force dipoles, geometry and intercellular interactions. Nor can the material nematic tensor in a constitutive stress law be replaced without validation by a tensor calculated from optical-flow directions: one describes organization of a material field, whereas the other describes image-derived kinematics.

Our evidentiary ranking is therefore explicit. Shape-density fluidity in patient-derived breast and cervix tissue has direct experimental support. Defect-controlled extrusion has strong support in non-cancer monolayers. Defect-guided tumor invasion and a universal mapping from molecular contractility to tissue active-stress sign remain hypotheses. This ranking should be updated as three-dimensional live measurements mature, not blurred by using the same terminology for all evidence levels.

BOX 3. **Why single-target migrastatics can fail.**

Migration inhibition can expose compensatory routes. Arp2/3 loss may be buffered by formin-dependent protrusion (Dimchev et al., 2021), and protease inhibition can favor protease-independent migration (Wolf et al., 2003). These findings support combination strategies in principle, but they do not identify a universally safe shared node. Actin turnover, myosin, adhesion and Rho-GTPase signaling are also required in normal epithelia, immune cells and vasculature. A rational migrastatic program must therefore measure both suppression of the intended invasion route and induction of an escape route, while testing normal-tissue and immune consequences. Mechanical observables are useful response variables for that purpose; they are not, by themselves, therapeutic targets.
